# DSCC1 interacts with HSP90AB1 and promotes the progression of lung adenocarcinoma via regulating ER stress

**DOI:** 10.1186/s12935-023-03047-w

**Published:** 2023-09-23

**Authors:** Xu Lin, Ye-han Liu, Huan-qi Zhang, Lin-wen Wu, Qi Li, Jun Deng, Qingyi Zhang, Yuhong Yang, Chong Zhang, Yang-ling Li, Jian Hu

**Affiliations:** 1 Department of Thoracic Surgery, The First Affiliated Hospital, Zhejiang University School of Medicine, Hangzhou, 310003 China; 2 School of Medicine, Hangzhou City University, No.51 Huzhou Street, Hangzhou, Zhejiang 310015 China; 3 College of Pharmaceutical Sciences, Zhejiang University, Hangzhou, 310058 China; 4 Key Laboratory of Clinical Cancer Pharmacology and Toxicology Research of Zhejiang Province, Affiliated Hangzhou First People’s Hospital, Zhejiang University School of Medicine, Hangzhou, 310006 China; 5 Department of Clinical Pharmacy, Affiliated Hangzhou First People’s Hospital, Zhejiang University School of Medicine, Hangzhou, 310006 China; 6 Cancer Center, Zhejiang University, Hangzhou, 310058 China

**Keywords:** DSCC1, HSP90AB1, Lung adenocarcinoma, PD-L1, Proliferation, Stemness, Metastasis

## Abstract

Lung cancer is a leading cause of cancer-related deaths, and the most common type is lung adenocarcinoma (LUAD). LUAD is frequently diagnosed in people who never smoked, patients are always diagnosed at advanced inoperable stages, and the prognosis is ultimately poor. Thus, there is an urgent need for the development of novel targeted therapeutics to suppress LUAD progression. In this study, we demonstrated that the expression of DNA replication and sister chromatid cohesion 1 (DSCC1) was higher in LUAD samples than normal tissues, and the overexpression of DSCC1 or its coexpressed genes were highly correlated with poor outcomes of LUAD patients, highlighting DSCC1 might be involved in LUAD progression. Furthermore, the expression of DSCC1 was positively correlated with multiple genetic mutations which drive cancer development, including TP53, TTN, CSMD, and etc. More importantly, DSCC1 could promote the cell proliferation, stemness, EMT, and metastatic potential of LUAD cells. In addition, DSCC1 interacted with HSP90AB1 and promoted the progression of LUAD via regulating ER stress. Meanwhile, DSCC1 expression negatively correlated with immune cell infiltration in lung cancer, and DSCC1 positively regulated the expression of PD-L1 in LUAD cells. Collectively, this study revealed that DSCC1 is a novel therapeutic target to treat LUAD and a biomarker for predicting the efficiency of PD-1/PD-L1 blockade treatment.

**Supplementary Information:**

The online version contains supplementary material available at 10.1186/s12935-023-03047-w.

## Introduction

Lung cancer, one of the most frequently diagnosed cancers, is a leading cause of cancer-related deaths worldwide [1]. Non-small cell lung cancer (NSCLC) accounts for more than 80% of lung malignancies and encompasses lung adenocarcinoma (LUAD), squamous cell cancer (LUSC), and large cell cancer [2, 3]. Among three NSCLC subtypes, LUAD is the most common type of NSCLC accounting for approximately 40–50% of lung cancer [4]. Because LUAD is frequently to be diagnosed in people who never smoked, the early diagnosis of LUAD is particularly challenging [5]. Thus, multiple LUAD patients are diagnosed at advanced inoperable stages, and the prognosis of advanced LUAD is ultimately poor [6]. The prognosis of LUAD patients has been gradually elevated due to the development of targeted molecular therapy against driver gene aberrations and immune checkpoint inhibition-based immunotherapy [7, 8]. However, multiple lung cancer patients cannot achieve an adequate response to these therapies [9]. Thus, novel therapeutics strategies are urgently needed in treating LUAD.

The endoplasmic reticulum (ER) is essential for protein synthesis and secretion, misfolded proteins accumulated in ER lumen results in ER stress, and ER stress induces the unfolded protein response (UPR) via upregulating chaperones, foldases, and proteins ensuring correct post-translational modifications [10]. ER stress is activated in multiple physiological or pathological conditions, for example, the treatment of chemotherapeutic agents, hormone therapy, and targeted therapies for cancer [11]. ER stress involves PERK activation which phosphorylates the α-subunit of the eukaryotic translation initiation factor 2 (eIF2α), eIF2α phosphorylation enhances ATF4 translation and thereby attenuates general protein translation to relieve the ER stress [12]. ER stress response pathways influence multiple tumorigenic and immune-regulatory molecules to dictate cancer progression, antitumor immunity and response to treatment [13]. The activation of UPR and ER stress can trigger either cancer cell survival or death, depending on duration and intensity of the stress [14].

DNA Replication and Sister Chromatid Cohesion 1 (DSCC1), the most important molecular to form a chromosome transmission-fidelity protein 18 (CTF18)-DSCC1-CTF8 (CTF18-1-8) module, is highly corelated with the growth and metastasis of colon or prostate cancer cells [15–18]. In addition, DSCC1 is also considered as a potential diagnostic and prognostic biomarker for breast carcinoma, and facilitates the progression of breast carcinoma by activating Wnt/β-catenin signaling and inhibiting p53 [19]. Furthermore, DSCC1 promotes proliferation and is associated with poor prognosis of hepatocellular carcinoma [20]. Meanwhile, DSCC1 is considered as a potential diagnostic molecule for LUAD, and high DSCC1 predicts poor prognosis of LUAD [21]. However, the role and mechanism of DSCC1 in lung cancer progression remain unclear. In this study, we firstly demonstrated that DSCC1 regulates ER stress and promotes LUAD progression via interacting with HSP90AB1, and DSCC1 might be a novel therapeutic target for LUAD.

## Materials & methods

### Cell culture and transfection

A549, NCI-H1299 and NCI-H460 cells were obtained from Cell Bank of Type Culture Collection of Chinese Academy of Science. All these cells were maintained with RPMI-1640 medium plus 10% FBS. Jet-PRIME transfection reagents (Polyplus transfection) were applied to transfect plasmids or siRNAs following the instructions from manufacturer. The siRNA targeting human DSCC1 and HSP90AB1 were obtained from GenePharma Corporation (Shanghai, China). Overexpression plasmids targeting human DSCC1 were purchased from GenScript (Nanjing, China). The sense sequences of the DSCC1 siRNAs were 5′-GCCUGUAAGAUUGGAGGUUTT-3′ (DSCC1 siRNA-1) and 5′-GGACCAGUUGAAGAAGGAATT-3′ (DSCC1 siRNA-2), that of the HSP90AB1 siRNA was 5′- GCUUCGAGGUGGUAUAUAUTT-3′, and that of the negative control siRNA was 5′-UUCUCCGAACGUGUCACGUTT-3′.

### Western blot and immunoprecipitation

Western blot analysis and immunoprecipitation were performed as previously reported [22]. Antibodies against DSCC1 (HPA024401) was purchased from The Human Protein Altas (Sweden). Antibodies against phospho-Smad3 (Ser423/425) (#9520), Jak2 (#3230) and phospho-eIF2α (#3398S) were purchased from Cell Signaling Technology (Danvers, MA, USA). Antibodies against STAT3 (sc-482), Smad2/3 (sc-133,098) and β-Actin (sc-47,778) were obtained from Santa Cruz Biotechnology (Santa Cruz, CA, USA). The anti-phospho-STAT3 (Tyr705) (ab76315) and anti-programmed death ligand-1 (PD-L1, ab205921) antibodies were purchased from Abcam (Cambridge, Cambridgeshire, UK). Antibodies against SOX2 (11064-1-AP), NANOG (14295-1-AP), OCT4 (11263-1-AP), E-cadherin (20874-1-AP), N-cadherin (22018-1-AP), ATF4 (10835-1-AP), HSP90AB1 (11405-1-AP), Vimentin (10366-1-AP), Vinculin (66305-1-Ig) were supplied by Proteintech (Wuhan, China). An anti-GAPDH antibody (db106) was supplied by Diagbio (Hangzhou, China).

### LC-ESIMS/MS analysis by Q Exactive HF

The proteins pulled down from IP were separated using SDS-PAGE and identified by LC-ESIMS/MS analysis (Micrometer Biotech Company, Hangzhou, China) [23]. Meanwhile, protein samples were collected for SDS-PAGE gel electrophoresis after immunoprecipitation, and silver staining experiment was performed using Fast Silver Stain Kit (Beyotime, P0017S).

### Quantitative PCR analysis

Quantitative PCR was performed as previously reported [22]. The sequences of the primers were listed in Supplementary Table 3.

### Cell migration and invasion assay

Indicated LUAD cells (5–10 × 10^4^) were seeded in top chambers of the transwell plates coated without or with Matrigel, respectively. The upper chamber of transwell was filled with serum-free culture medium and the lower chamber was filled with culture medium plus 10% FBS. The migratory or invasive cells were fixed by methanol, and incubated with 0.5% crystal violet.

### Wound-healing assay

The transfected LUAD cells were seeded on 6-well plates with the intensity of 4 × 10^5^ cells/well. A 10 µl pipette tip was applied to obtain the wound. The cells were cultured with the serum-free RMPI-1640 after the scratch.

### Sulforhodamine B (SRB) and colony formation assays

The proliferation of LUAD cells was determined by the SRB assay as described. LUAD cells were transfected with DSCC1 siRNA or control siRNA and seeded on a 96-well plate in triplicate for indicated times. Meanwhile, the transfected cells were seeded on the 6-well plates and cultured for 12 days in colony formation assays. Cell colonies were fixed with methanol and then stained with crystal violet.

### Statistics

Data are shown as mean ± SD derived from three independent experiments. Comparison of 2 groups composed of continuous data was analyzed by 2-tailed Student’s t test. P values < 0.05 were considered significant. *P < 0.05; **P < 0.01; ***P < 0.001.

## Results

### Overexpression of DSCC1 predicts poor outcomes of LUAD patients

**Fig. 1:**
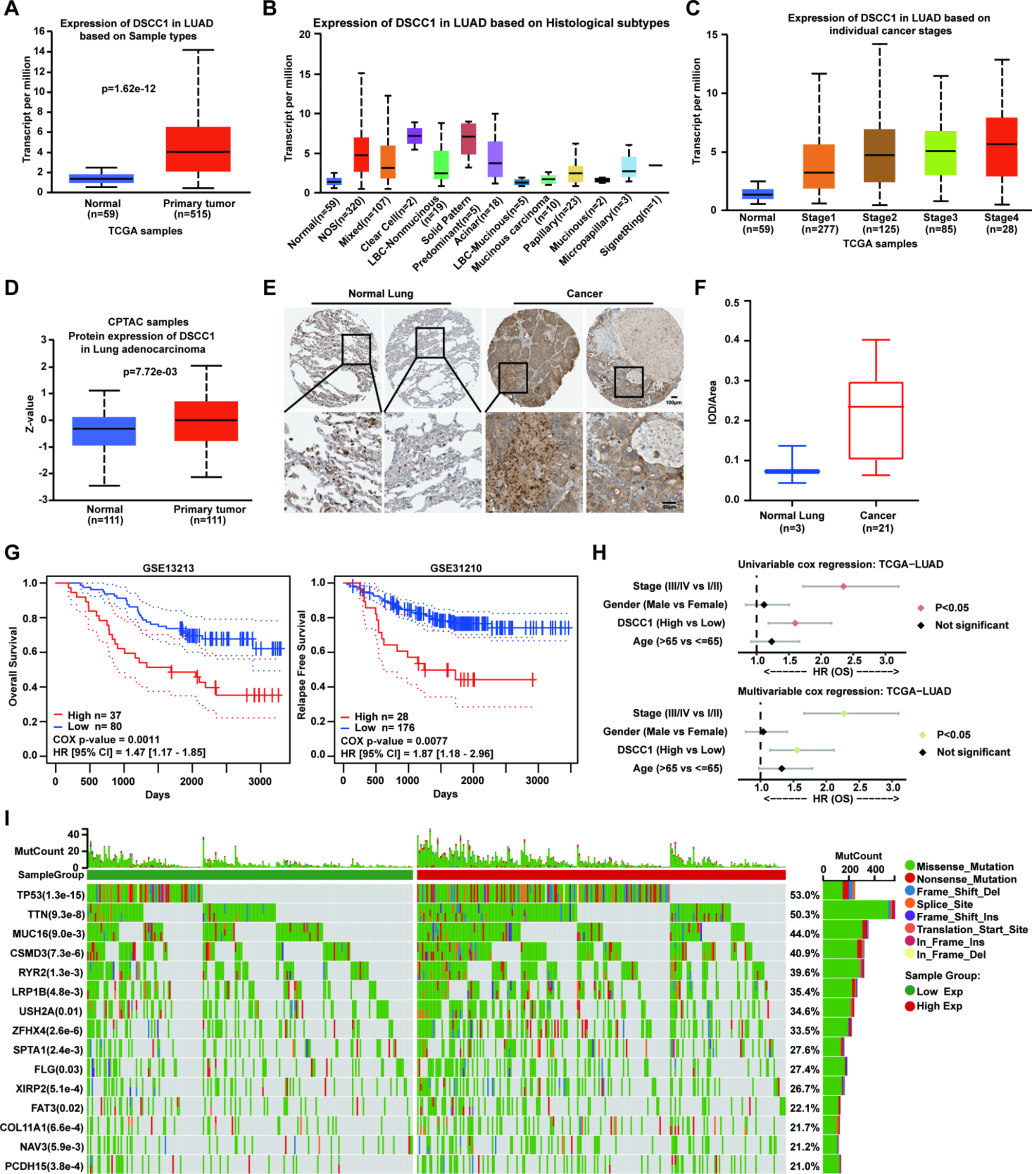
DSCC1 is overexpressed in LUAD and predicts poor outcome of LUAD patients. **(A-D)** The data was obtained from UALCAN (http://ualcan.path.uab.edu/index.html). TCGA; Gene: DSCC1; TCGA dataset: LUAD; Expression; DSCC1 expression based on Sample type **(A)**, Tumor histology **(B)**, individual cancer stages **(C)**. CPTAC; Gene: DSCC1; CPTAC dataset: LUAD; Total protein **(D)**. **(E,F)** The data was obtained from The Human Protein Atlas (https://www.proteinatlas.org/). **(G)** The results were collected from PrognoScan (http://dna00.bio.kyutech.ac.jp/PrognoScan/index.html). Overall Survival: Dataset - GSE13213 (left panel); Relapse Free Survival: Dataset - GSE31210 (right panel). **(H)** The data was collected from CAMOIP (https://www.camoip.net/). COX Regression; Gene expression; TCGA-Cohort: LUAD-OS; Gene: DSCC1. **(I)** The data was obtained from Sangerbox 3.0 (http://sangerbox.com/home.html). Gene expression and mutation landscape; Gene: DSCC1; Cancer: LUAD.

As shown in Fig. 1A, the mRNA level of DSCC1 was overexpressed in LUAD samples compared with normal tissues (p = 1.62e-12) [24]. Moreover, the overexpression of DSCC1 was observed in multiple histological subtypes of LUAD sample in comparison of normal tissues (Fig. 1B). The mRNA level of DSCC1 increased accompanied by LUAD development from stage 1 to stage 4 (Fig. 1C). Meanwhile, the expression of DSCC1 protein was also overexpressed in LUAD sample as compared to normal tissues (Fig. 1D) [25]. Furthermore, the overexpression of DSCC1 could also be observed in LUAD as compared with normal tissue using immunohistochemistry from The Human Protein Atlas (Fig. 1E,F and Supplementary Fig. 1) [26, 27]. In addition, DSCC1 overexpression predicted poor overall survival and relapse free survival of LUAD patients (Fig. 1G) [28]. Besides the univariable COX regression analysis, multivariate COX regression analysis also demonstrated that the expression of DSCC1 was also an independent prognostic factor (HR = 1.56, p = 0.00486), in addition to stage (HR = 2.27, p < 0.05) in LUAD patients (Fig. 1H) [29]. More interestingly, the expression of DSCC1 was positively correlated with multiple genetic mutations which drive cancer development, including TP53 (p = 1.3e-15), TTN (p = 9.3e-8), CSMD (p = 7.3e-6), ZFHX4 (p = 2.6e-6), COL11A1 (p = 6.6e-4), PCDH15 (p = 3.8e-4), XIRP2 (p = 5.1e-4), NAV3 (p = 5.9e-3), RYR2 (p = 1.3e-3), SPTA1(p = 2.4e-3), LRP1B (p = 4.8e-3), MUC16 (p = 9.0e-3) mutations (Fig. 1I) [30]. Overall, we hypothesized that DSCC1 might be involved in LUAD development.

### The overexpression of DSCC1 and its coexpressed genes predict poor outcomes in LUAD patients

**Fig. 2:**
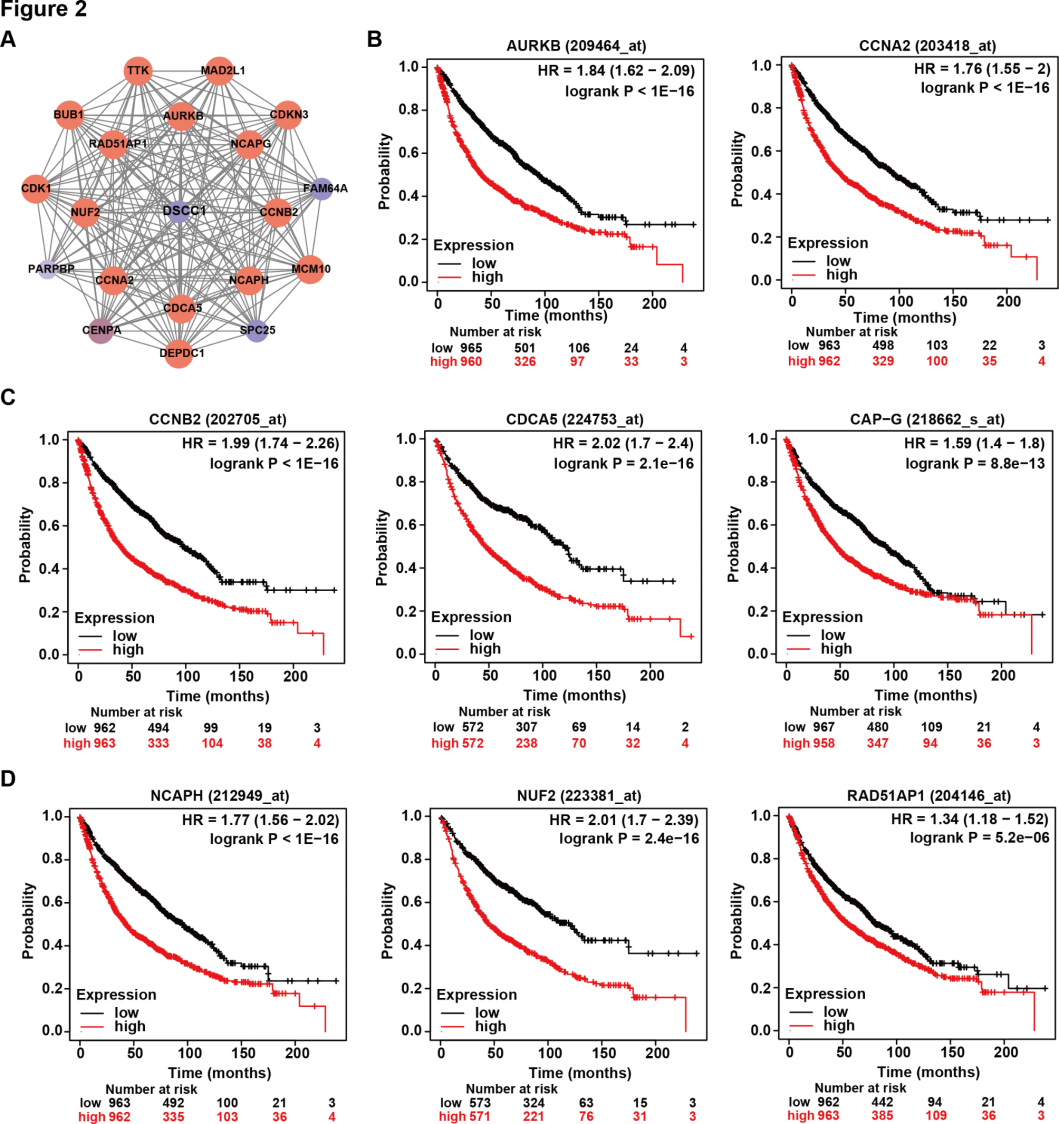
The high levels of DSCC1 coexpressed genes predict poor outcomes of LUAD patients. **(A)** The coexpressed genes of DSCC1 were obtained from LinkedOmics (http://www.linkedomics.org/admin.php). TOP 20 genes were identified to construct a protein-protein interaction network using STRING (https://www.string-db.org/). (B,C,D) The results were collected from KM plotter (http://kmplot.com/analysis/index.php?p=background). The overall survival times of lung cancer patients were shown (OS, n = 1927)

In order to further investigate the prognostic value of DSCC1 correlated genes in LUAD patients, we identified DSCC1 coexpressed genes in clinical LUAD samples using LinkedOmics [31]. Next, TOP 20 coexpressed genes that had positive correlation with DSCC1 were identified to construct a protein-protein interaction network using STRING analysis followed by Cytoscape (Fig. 2A) [32, 33]. As shown in Fig. 2B-D, the high levels of DSCC1 coexpressed genes were correlated with poor clinical outcome of LUAD patients (AURKB: hazard ratio (HR) [95% CI] = 1.84 [1.62–2.09], P < 1e-16; CCNA2: HR [95% CI] = 1.76 [1.55-2.00], P < 1e-16; CCNB2: HR [95% CI] = 1.99 [1.74–2.26], P < 1e-16; CDCA5: HR [95% CI] = 2.02 [1.70–2.40], P = 2.1e-16; CAPG: HR [95% CI] = 1.59 [1.40–1.80], P = 8.8e-13; NCAPH: HR [95% CI] = 1.77 [1.56–2.02], P < 1e-16; NUF2: HR [95% CI] = 2.01 [1.70–2.39], P = 2.4e-16; RAD51AP1: HR [95% CI] = 1.34 [1.18–1.52], P = 5.2e-6). Thus, patients harboring higher level of DSCC1-coexpressed genes in LUAD predicted worse prognosis compared with those LUAD patients harboring low level of DSCC1.

### DSCC1 promotes LUAD cell proliferation and stemness

**Fig. 3:**
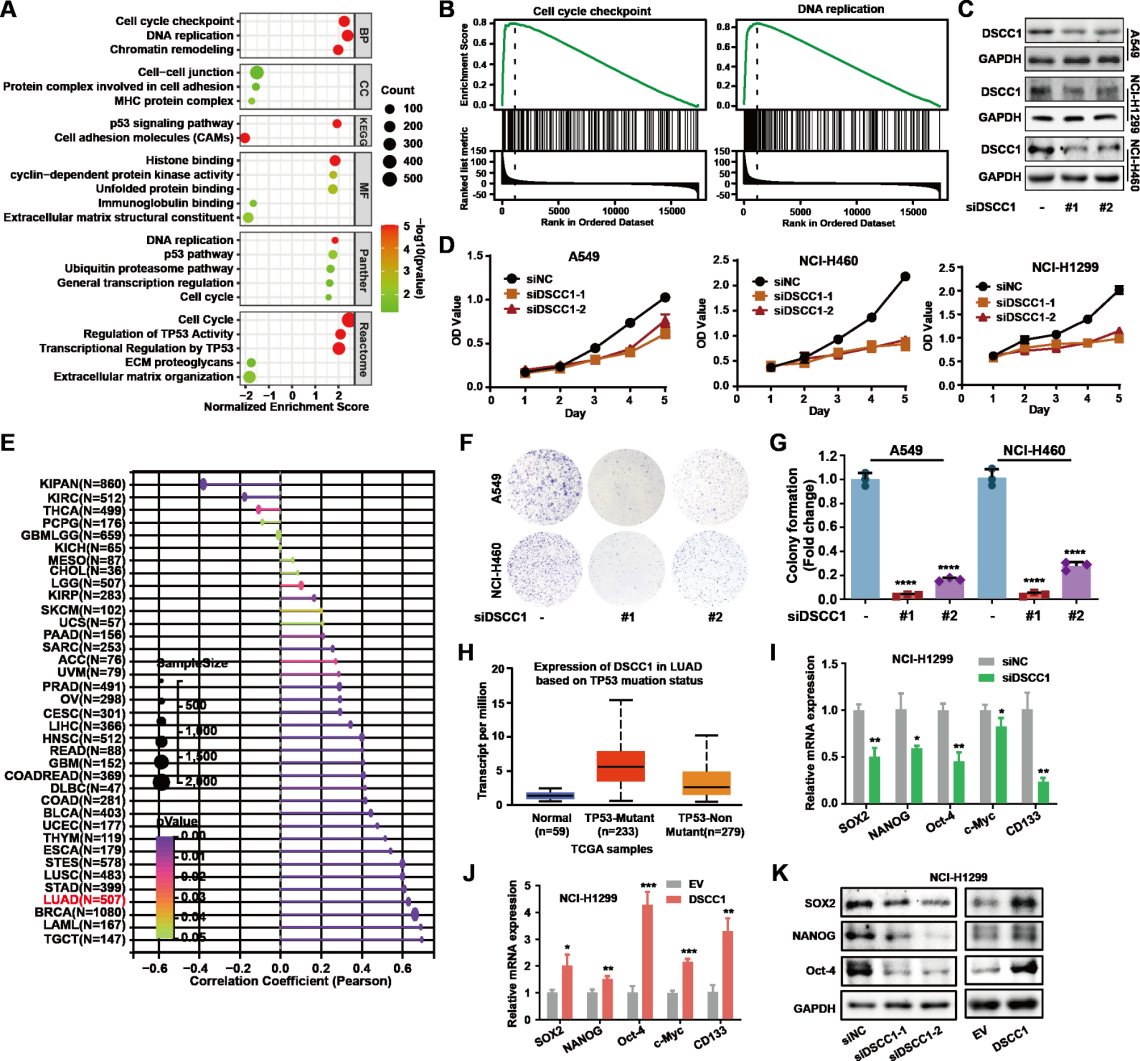
DSCC1 promotes LUAD cell proliferation and stemness. **(A-B)** The data was collected from LinkedOmics. Gene: DSCC1; Sample cohort: TCGA_LUAD; Institute: UNC; Data type: RNA seq; Platform: HiSeq RNA; Statistical Method: Pearson correlation test; Patients: 515; Enrichment Analysis: GSEA; Rank Criteria (from LinkFinder Result): FDR. **(C)** LUAD cells were transfected with control siRNA or siDSCC1 for 48 h, and the expression of DSCC1 was determined. **(D)** LUAD cells were transfected with control siRNA or siDSCC1 for the indicated times, and SRB was used to detect cell proliferation. **(E)** The data was obtained from Sangerbox 3.0 (http://sangerbox.com/home.html). Cancer stemness and gene expression: Gene: DSCC1; Data source: RNAss; Data transformation: log2(x + 0.001); Pearson. **(F,G)** LUAD cells were transfected with DSCC1 siRNA and control siRNA and the colony formation was performed. **(H)** The data was obtained from UALCAN. **(I)** LUAD cells were transfected with DSCC1 siRNA and control siRNA for 48 h, and the mRNA level of indicated proteins were detected by quantitative PCR analysis. **(J)** LUAD cells were transfected with DSCC1 overexpressing plasmid and empty vector for 48 h, and the mRNA level of indicated proteins were detected by quantitative PCR analysis. **(K)** LUAD cells were transfected with DSCC1 siRNA and control siRNA for 48 h, and the expression of indicated proteins were detected by western blot

In order to determine the function of DSCC1 in promoting LUAD development, GSEA enrichment was performed by LinkedOmics online tool [31]. GSEA enrichment demonstrated that DSCC1 was positively correlated with cell cycle, cyclin-dependent protein kinase activity, cell cycle check point, and DNA replication (Fig. 3A and B). These data suggested that DSCC1 might be involved in cell proliferation of LUAD. Indeed, DSCC1 silence significantly suppressed the proliferation of LUAD cells (Fig. 3C and D, Supplementary Fig. 2A). In addition, the expression of DSCC1 was closely correlated with the features of cancer stem cells of LUAD (Fig. 3E). Indeed, DSCC1 knockdown significantly suppressed the colony formation of LUAD cells (Fig. 3F and G). p53 mutations have been observed in up to 50% of all human cancers and induce an enhancement in oncogenic phenotypes such as proliferation and tumorigenicity [34]. GSEA enrichment demonstrated that DSCC1 was positively correlated with regulation of TP53 activity, transcriptional regulation by TP53, and p53 pathway. Meanwhile, DSCC1 is overexpressed in TP53 mutant LUAD samples compared with TP53 nonmutant LUAD samples (Fig. 3H, P = 8.03E-9). Furthermore, DSCC1 silence could downregulate the expression of multiple biomarkers of stem-like tumor cells, including SOX2, NANOG, and Oct-4 (Fig. 3I). In contrast, DSCC1 overexpression could upregulate biomarkers of stem-like tumor cells in LUAD cells (Fig. 3J). Thus, DSCC1 promoted the stemness of LUAD cells via regulating SOX2, NANOG, and Oct-4 (Fig. 3K). These results suggested that DSCC1 reinforced the cell proliferation and stemness of LUAD cells.

### DSCC1 promotes the metastatic potential of LUAD cells

**Fig. 4:**
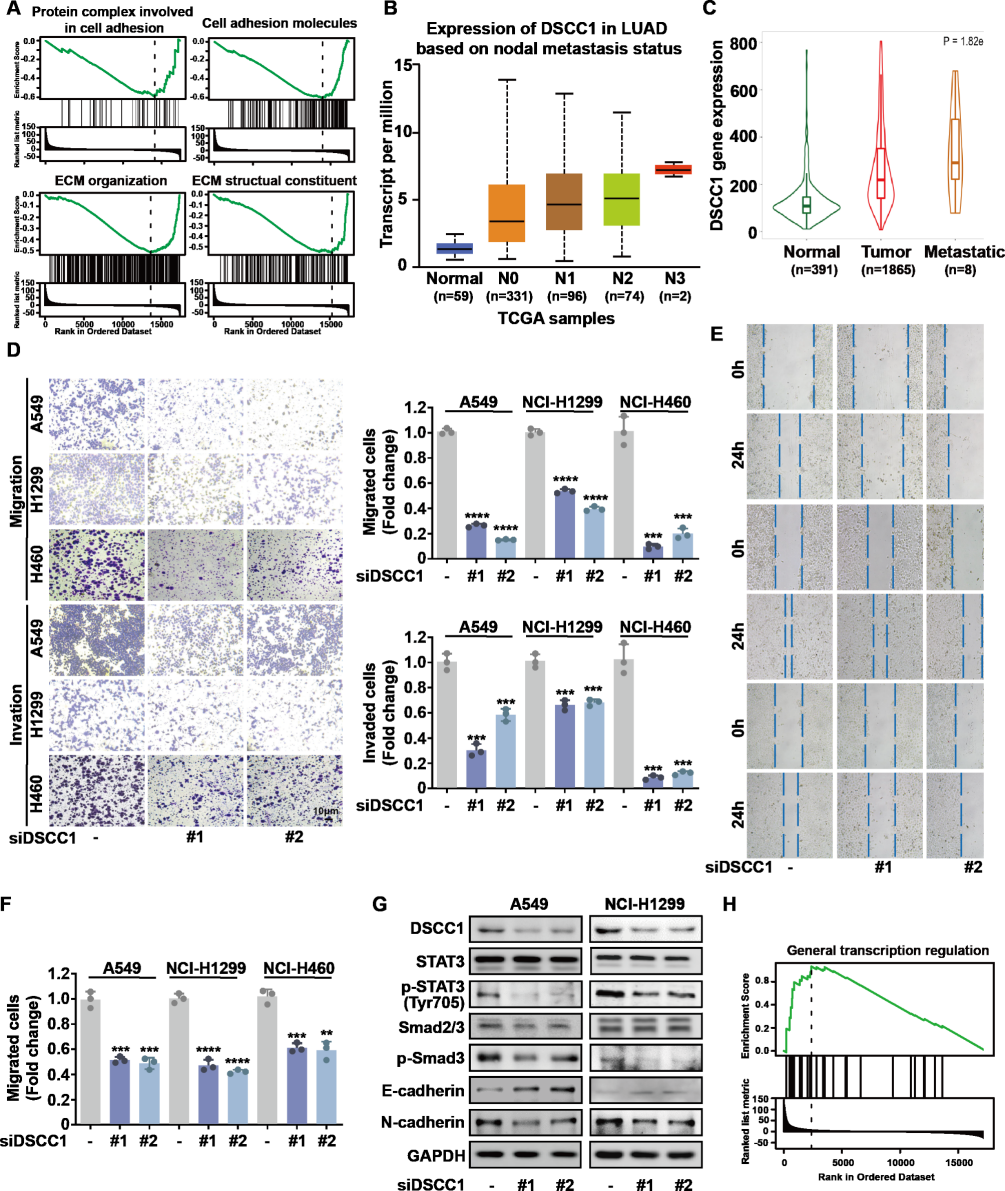
DSCC1 siRNA suppresses the metastatic potential of LUAD cells. **(A)** The data was collected from LinkedOmics. **(B)** The data was obtained from UALCAN. **(C)** The result was collected from TNMplot (https://tnmplot.com/analysis/). Gene expression comparison: compare tumor, normal and metastasis; Gene chip data; Gene: DSCC1; Tissue: Lung. **(D)** LUAD cells were transfected with DSCC1 siRNA and control siRNA for 48 h, and the migrative and invasive abilities of LUAD cells were determined by Transwell assay. **(E-F)** LUAD cells were transfected with DSCC1 siRNA and control siRNA for 24 h, and the migration was determined by wound healing assay. **(G)** LUAD cells were transfected with DSCC1 siRNA and control siRNA for 48 h, and the expression of indicated proteins were detected by western blot. **(H)** The data was collected from LinkedOmics.

GSEA enrichment showed that DSCC1 was negatively correlated with cell-cell junction, protein complex involved in cell adhesion, cell adhesion molecules (CAMs), extracellular matrix (ECM) structural constituent, ECM proteoglycans, and ECM organization (Fig. 3 A). Furthermore, the expression of DSCC1 increased with incidence of LUAD nodal metastasis (Fig. 4B). Meanwhile, DSCC1 was significantly overexpressed in the metastatic tumors compared with LUAD primary tumors (Fig. 4C) [35]. Thus, we hypothesized that DSCC1 might promote the metastatic potential of LUAD cells. Indeed, DSCC1 silence significantly suppressed the migrative and invasive abilities of LUAD cells (Fig. 4D). Meanwhile, wound healing assay also demonstrated that DSCC1 knockdown inhibited the migration of LUAD cells (Fig. 4E F). Epithelial-mesenchymal transition (EMT) is a vital step in the early stages of cancer metastasis and orchestrated by multiple signaling pathways, such as IL-6/JAK/STAT3 and TGF-β/Smad signaling pathway [22]. DSCC1 siRNA restrained the progress of EMT via upregulating E-cadherin and downregulating N-cadherin in LUAD cells (Fig. 4G). In addition, GSEA enrichment demonstrated that DSCC1 expression was positively corelated with general transcription regulation in LUAD samples (Fig. 4H). Indeed, the depletion of DSCC1 markedly repressed the activation of STAT3 and Smad2/3 signaling pathway (Fig. 4G).

**Fig. 5:**
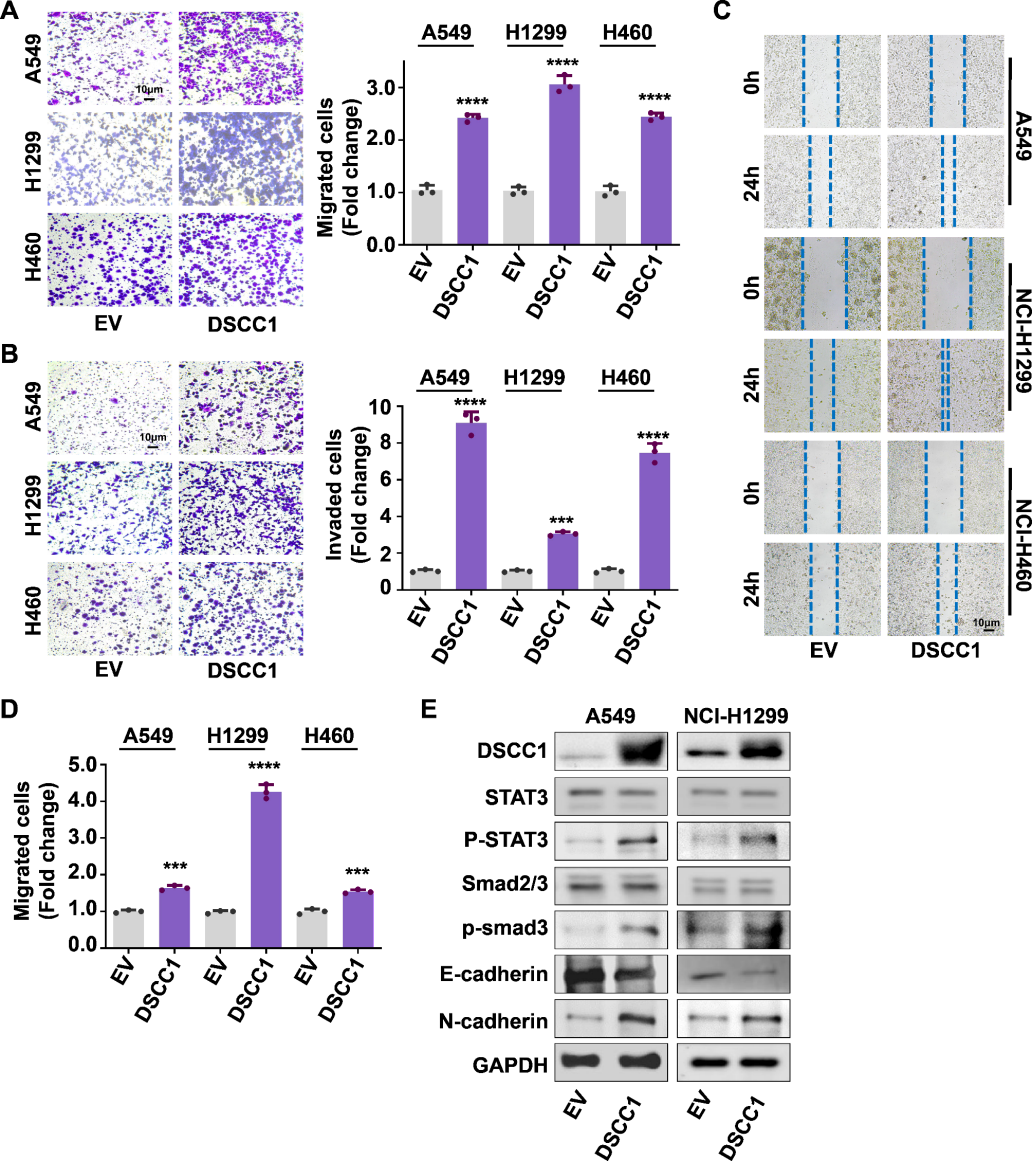
DSCC1 promotes the metastatic potential of LUAD cells. **(A-B)** LUAD cells were transfected with DSCC1 overexpressing plasmid and empty vector for 48 h, and the migrative and invasive abilities of LUAD cells were determined by Transwell assay. **(C-D)** LUAD cells were transfected with DSCC1 overexpressing plasmid and empty vector for 24 h, and the migration was determined by wound healing assay. **(E)** LUAD cells were transfected with DSCC1 overexpressing plasmid and empty vector for 48 h, and the expression of indicated proteins were detected by western blot

In contrast, DSCC1 overexpression significantly reinforced the migrative and invasive abilities of LUAD cells (Fig. 5A and B). Meanwhile, wound healing assay also confirmed that the overexpression of DSCC1 enhanced the migration of LUAD cells (Fig. 5C and D). In addition, DSCC1 induced EMT progress and the activation of STAT3 and Smad2/3 signaling pathway in LUAD cells (Fig. 5E). Collectively, these data suggested that DSCC1 promoted EMT and the metastatic potential of LUAD cells via regulating STAT3 and Smad2/3 pathways.

### DSCC1 interacts with HSP90AB1 and promotes the progression of LUAD

**Fig. 6:**
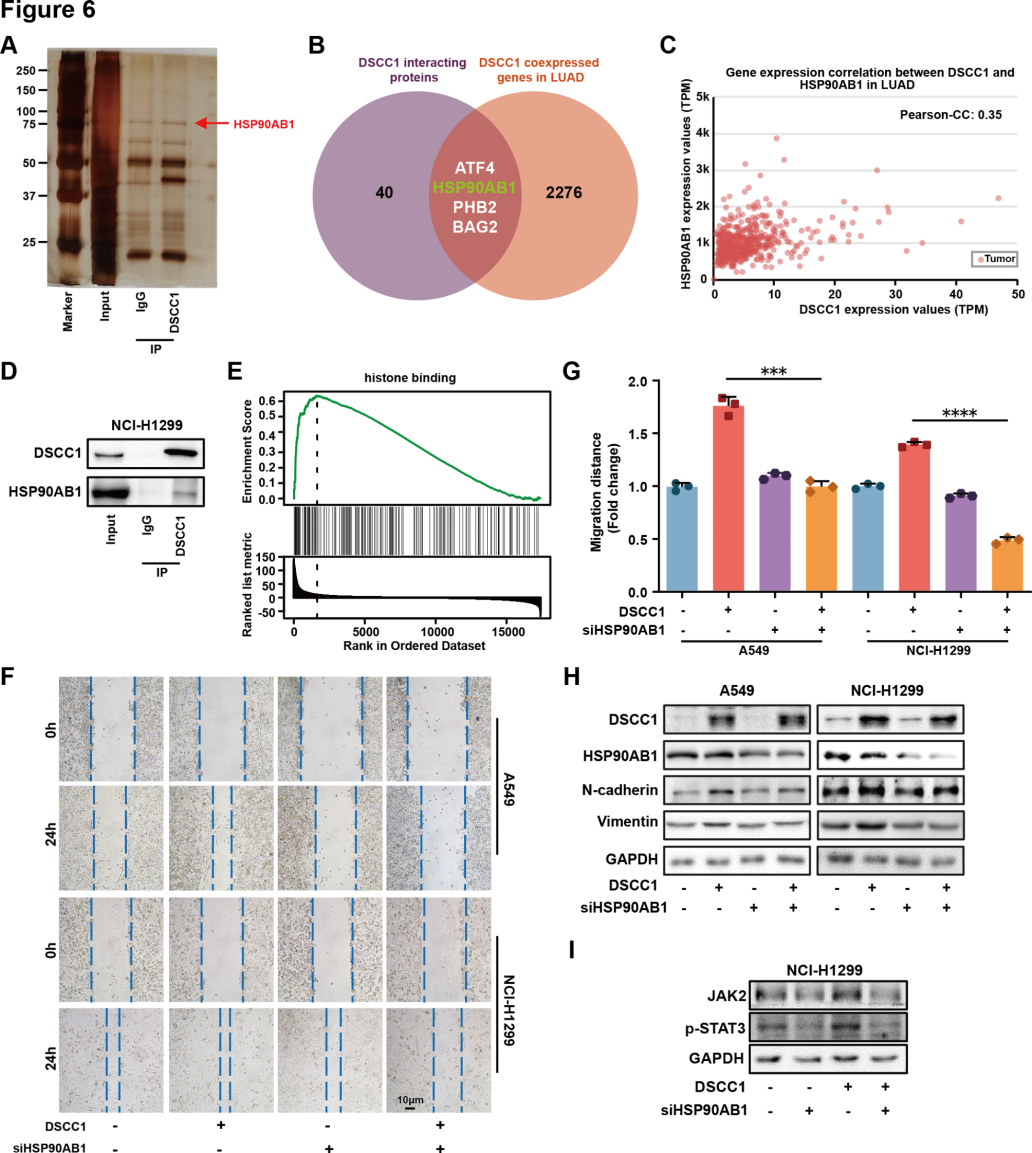
DSCC1 interacts with HSP90AB1 and promotes the progression of LUAD. **(A)** LUAD cells were collected, and immunoprecipitations with DSCC1 antibody or IgG were performed. DSCC1 interacting proteins were identified by immunoprecipitation coupled to mass spectrometry. **(B-C)** DSCC1 coexpressed genes were identified by UALCAN platform, and A Venn diagram was generated by overlapping DSCC1 coexpressed genes and DSCC1 interacting proteins. **(D)** Immunoprecipitation was performed to verify the interaction between DSCC1 and HSP90AB1. **(E)** The data was obtained from LinkedOmics. **(F-G)** LUAD cells with high level of DSCC1 were transfected with control siRNA and HSP90AB1 siRNA for 24 h, and migrative abilities of LUAD cells were determined by wound healing assay. **(H-I)** LUAD cells with high level of DSCC1 were transfected with control siRNA and HSP90AB1 siRNA for 48 h, and the expression of the indicated proteins were detected by western blot

In order to investigate the mechanism of DSCC1 in promoting cell proliferation and metastasis, the proteins interacting with DSCC1 were analyzed by immunoprecipitation coupled to mass spectrometry, and we identified 44 proteins which specifically interact with DSCC1 in LUAD cells (Fig. 6A and Supplementary Table 1). We also identified DSCC1-coexpressed genes in LUAD clinical samples with Pearson correlation coefficient (r) values greater than 0.3 using UALCAN (Supplementary Table 2). A Venn diagram was generated which showed that there were 4 overlapping genes between DSCC1-interacting and coexpressing proteins in LUAD, including ATF4, HSP90AB1, PHB2, and BAG2 (Fig. 6B). PHB2 and HSP90AB1 were overexpressed in the metastatic tumors compared with LUAD primary tumors, and high level of HSP90AB1 predicted shorter overall survival and first progression times of LUAD patients (Supplementary Fig. 2B and 2 C) [36]. Thus, we hypothesized that DSCC1 might interact with HSP90AB1 and promote LUAD progression. Indeed, DSCC1 and HSP90AB1 were coexpressed in LUAD clinical samples, and the interaction between DSCC1 and HSP90AB1 could be verified by immunoprecipitation (Fig. 6C and D). HSP90 binds histones H1, H2A, H2B, H3 and H4 with high affinity and influences transcription by modulating histone modification during mitosis, or after cellular stress [37, 38]. Most importantly, GSEA enrichment showed that DSCC1 was positively correlated with histone binding in LUAD, indicating that DSCC1 might interact with HSP90AB1 and regulate transcription by binding with histones (Fig. 6E). In addition, HSP90AB1 silence significantly suppressed the enhanced migrative ability induced by DSCC1 overexpression (Fig. 6F and G). Meanwhile, HSP90AB1 knockdown reversed DSCC1-activated EMT and STAT3 in LUAD cells (Fig. 6H and I). These results suggested that DSCC1 might promote LUAD progression via interacting with HSP90AB1 and regulating STAT3 transcription.

### DSCC1 promotes LUAD progression via regulating ER stress and HSP90AB1

**Fig. 7:**
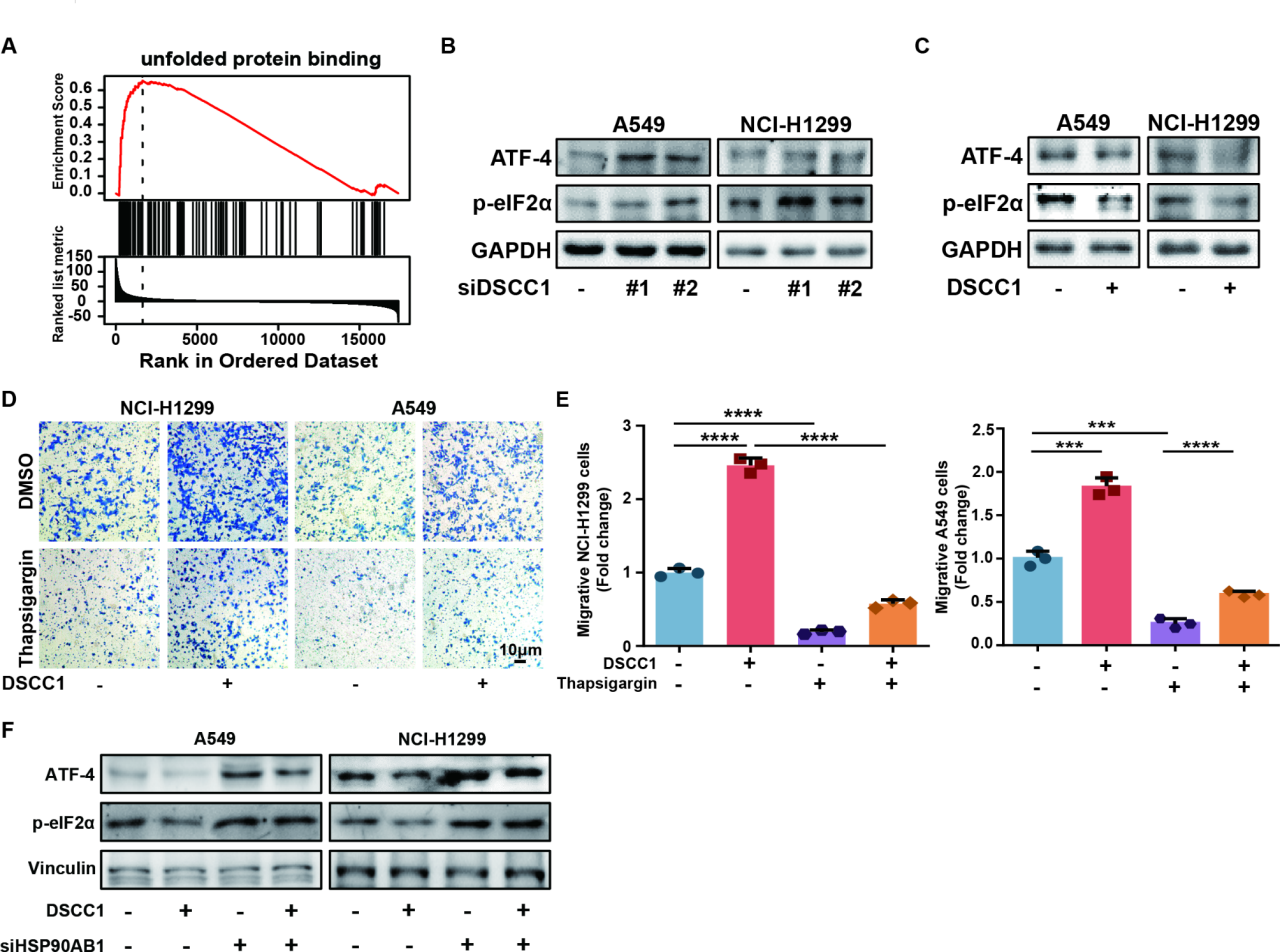
DSCC1 promotes LUAD progression via regulating ER stress and HSP90AB1. **(A)** The data was obtained from LinkedOmics. **(B)** LUAD cells were treated with control siRNA and DSCC1 siRNA for 48 h, and the expression of the indicated proteins were detected by western blot. **(C)** LUAD cells were treated with DSCC1 overexpressing plasmid and empty vector for 48 h, and the expression of the indicated proteins were detected by western blot. **(D-E)** LUAD cells were transfected with DSCC1 plasmid and empty vector for 48 h, then treated with thapsigargin (100 nM for NCI-H1299, and 10 nM for A549) for 24 h, and the migrative ability of LUAD cells were detected. **(F)** LUAD cells overexpressing DSCC1 were transfected with control siRNA and HSP90AB1 siRNA for 48 h, and the expression of the indicated proteins were detected by western blot

GSEA enrichment showed that DSCC1 was positively correlated with unfolded protein binding (Fig. 7A). In addition, The HSP90 family members play critical role in UPR and ER stress, and multiple compounds can inhibit HSP90 and trigger ER stress in lung cancer cells [39, 40]. Thus, we hypothesized that DSCC1 might interact with HSP90AB1 and regulate ER stress. Indeed, DSCC1 silence reinforced the expression of ATF-4 and p-eIF2α, indicating that DSCC1 silence activated ER stress in LUAD cells (Fig. 7B). In contrast, DSCC1 overexpression suppressed the activation of ER stress in LUAD cells (Fig. 7C). Meanwhile, ER stress inducer thapsigargin successfully reversed the enhanced migrative ability of LUAD cells induced by DSCC1 overexpression (Fig. 7D and E). In addition, HSP90AB1 silence activated ER stress and enhanced the suppression of ER stress induced by DSCC1 in LUAD cells. Collectively, DSCC1 suppresses ER stress and promotes LUAD progression via regulating HSP90AB1.

### DSCC1 negatively correlated with tumor-infiltrating immune cells

**Fig. 8:**
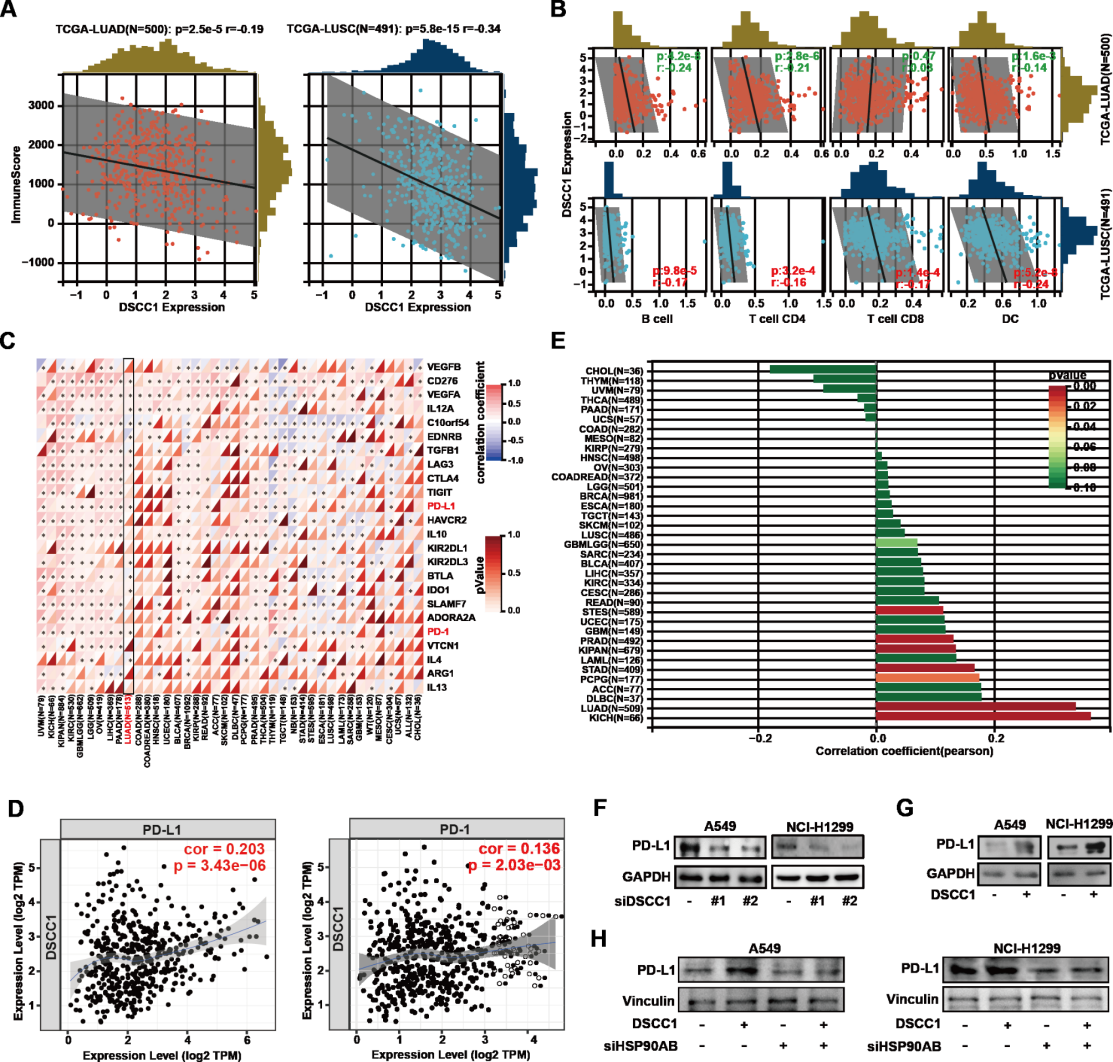
DSCC1 negatively correlated with tumor-infiltrating immune cells. **(A-C)** The data were collected from Sangerbox 3.0. Immune infiltration analysis, Immunocyte analysis (TIMER), and Immune checkpoint genes; Gene: DSCC1; Cancer: LUAD and LUSC. **(D)** The results were collected from TIMER (http://cistrome.org/TIMER/). **(E)** The data were collected from Sangerbox 3.0. **(F)** LUAD cells were transfected with DSCC1 siRNA and control siRNA for 48 h, and the expression of indicated proteins were determined. **(G)** LUAD cells were transfected with DSCC1 overexpressing plasmid and empty vector for 48 h, and the expression of indicated proteins were detected. **(H)** LUAD cells overexpressing DSCC1 were incubated with HSP90AB1 siRNA and control siRNA for 48 h, and the expression of indicated proteins were determined

The expression level of DSCC1 was negatively correlated with the immune score of lung cancer (Fig. 8A). Furthermore, DSCC1 expression correlated negatively with immune infiltration levels of multiple immune cell types in lung cancer and the proportions of B cells, CD4 + T cells, CD8 + T cells, and DC cells were lower in the high DSCC1 expression group than in the low DSCC1 expression group (Fig. 8B). In addition, the DSCC1 expression was positively correlated with multiple immune inhibitors in lung cancer, such as PD-L1 and programmed death-1 (PD-1) (Fig. 8C and D). Tumor mutational burden (TMB) and PD-L1 expression are most widely used immunotherapy biomarkers predicting response to checkpoint blockade in solid malignancies [41]. We demonstrated that the expression of DSCC1 was positively corelated with PD-L1, but also TMB in LUAD (Fig. 8E). We also verified the relationship between the expression of DSCC1 and PD-L1 in LUAD cells. As shown in Fig. 8F and G, DSCC1 silence significantly suppressed the expression of PD-L1, and overexpression of DSCC1 reinforced the level of PD-L1 in LUAD cells. Most importantly, HSP90AB1 silence reversed the expression of PD-L1 enhanced by DSCC1 overexpression in LUAD cells (Fig. 8H). These data suggested that DSCC1 might be a potential biomarker for predicting the efficacy of immunotherapy in LUAD treatment.

## Discussion

DSCC1 is involved in the cancer progression and its suppression may be a useful option for the treatment of multiple cancer, including colorectal cancer, breast cancer and hepatocellular carcinoma [17, 19, 20]. However, the role and mechanism of DSCC1 in the progression of lung cancer still need further investigation. In this study, we demonstrated that DSCC1 was overexpressed in LUAD samples compared with normal tissues, and the overexpression of DSCC1 and its coexpressed genes predicted poor outcomes of LUAD patients, highlighting DSCC1 might be involved in LUAD progression. More interestingly, we firstly showed that DSCC1 promoted the cell proliferation, stemness, EMT, and metastatic potential of LUAD cells. Furthermore, we also demonstrated that the expression of DSCC1 was positively correlated with multiple genetic mutations which drive cancer development, including TP53, TTN, CSMD, and etc. Thus, this study highlights that DSCC1 promotes cancer progression of LUAD, and DSCC1 suppression might be a novel therapeutics option for treating LUAD.

Heat-shock proteins are molecular chaperones which modify the structures and interactions of other proteins and can be divided in five subclasses, HSP90AA1, HSP90AA2, HSP90AB1, HSP90B1, and TRAP1 [42]. HSP90AB1 is upregulated in numerous solid tumors, and promotes the cancer progression via enhancing cancer cell proliferation, EMT, metastasis, and glycolysis [43, 44]. HSP90 members are molecular chaperones required for the stability and function of multiple signaling proteins that promote cancer progression [45]. For example, EEF1A2 acts as an oncogene and interacts with HSP90AB1 to promote LUAD metastasis via enhancing TGF-β/SMAD signaling [46]. In this study, we observed that DSCC1 interacted and coexpressed with HSP90AB1 in LUAD which promoted cancer progression, indicating that HSP90AB1 might act as a molecular chaperone and be involved in DSCC1-promoted LUAD progression. HSP90 inhibition induces ER-stress mediated apoptosis in human cancer cells [47]. This study demonstrated that DSCC1 cooperated with HSP90AB1 and suppressed the activation of ER stress, and DSCC1 silence could successfully activated ER stress in LUAD cells. Collectively, DSCC1 interacts with HSP90AB1 and promotes the progression of LUAD via regulating ER stress. Furthermore, HSP90 is essential for the activation of JAK-STAT signaling [48]. Meanwhile, STAT3 localizes to the ER, acting as a gatekeeper for ER-mitochondrion Ca^2+^ fluxes and apoptotic responses, and STAT3 silencing enhances ER Ca^2+^ release and sensitivity to apoptosis following oxidative stress [49]. Thus, we hypothesized that DSCC1 might interact with HSP90AB1 and regulate ER stress via JAK-STAT signaling. Indeed, DSCC1 induced the activation of STAT3, and HSP90AB1 silence reversed DSCC1-activated STAT3 in LUAD cells, indicating that DSCC1 activated STAT3 via HSP90AB1. Thus, our data explained the mechanism underlying DSCC1-promoted LUAD progression.

Cancer immunotherapy targeting the PD-1/PD-L1 pathway is clinically beneficial and widely used in lung cancer patients [50]. Despite the great progress in immunotherapy for lung cancer patients, only a small proportion of patients show a good response to immunotherapy [51]. Thus, it is important to uncover predictive biomarkers providing clinicians with useful information to concern patient prognosis and therapeutic efficacy for PD-1/PD-L1 blockade treatment [52]. Although PD-L1 expression is an imperfect biomarker for PD-1/PD-L1 blockade treatment, it is the only biomarker recommended by the National Comprehensive Cancer Network guidelines to help making treatment decisions in metastatic lung cancer [53]. In this study, we found that DSCC1 was negatively correlated with tumor-infiltrating immune cells. More importantly, the DSCC1 expression was positively correlated with PD-L1 or PD-1 in lung cancer. These data firstly indicated that DSCC1 might be involved in tumor-induced immunosuppression during the progression of LUAD and a potential biomarker for predicting therapeutic efficacy of PD-1/PD-L1 blockade treatment. Meanwhile, the effectiveness of PD-1/PD-L1 blockade is also correlated with the TMB in lung cancer patients [54]. Our data suggested that DSCC1 expression was not only positively corelated with PD-L1/PD-1, but also TMB in lung cancer. Because PD-L1 expression cannot be used to accurately select patients for PD-1/PD-L1 blockade due to the low prediction accuracy, tumor-infiltrating immune cells and molecules in the tumor microenvironment along with PD-L1 expression may be important in predicting clinical benefits of PD-1/PD-L1 checkpoint blockades [55]. Our data indicated that DSCC1 expression was corelated with PD-1/PD-L1 expression, tumor immune infiltration, and TMB in lung cancer. Furthermore, TP53 somatic mutations are correlated with poor survival in NSCLC patients who undergo immunotherapy [56]. This study showed that DSCC1 was overexpressed in TP53 mutation LUAD samples compared with TP53 nonmutant samples, highlighting that DSCC1 expression might be corelated with therapeutic efficacy of immunotherapy. Thus, we hypothesized that DSCC1 expression might be a more efficiency biomarker for predicting the clinical outcomes of PD-1/PD-L1 blockade treatment. However, the clinical investigation is still needed to verify hypothesis.

## Conclusions

**Fig. 9:**
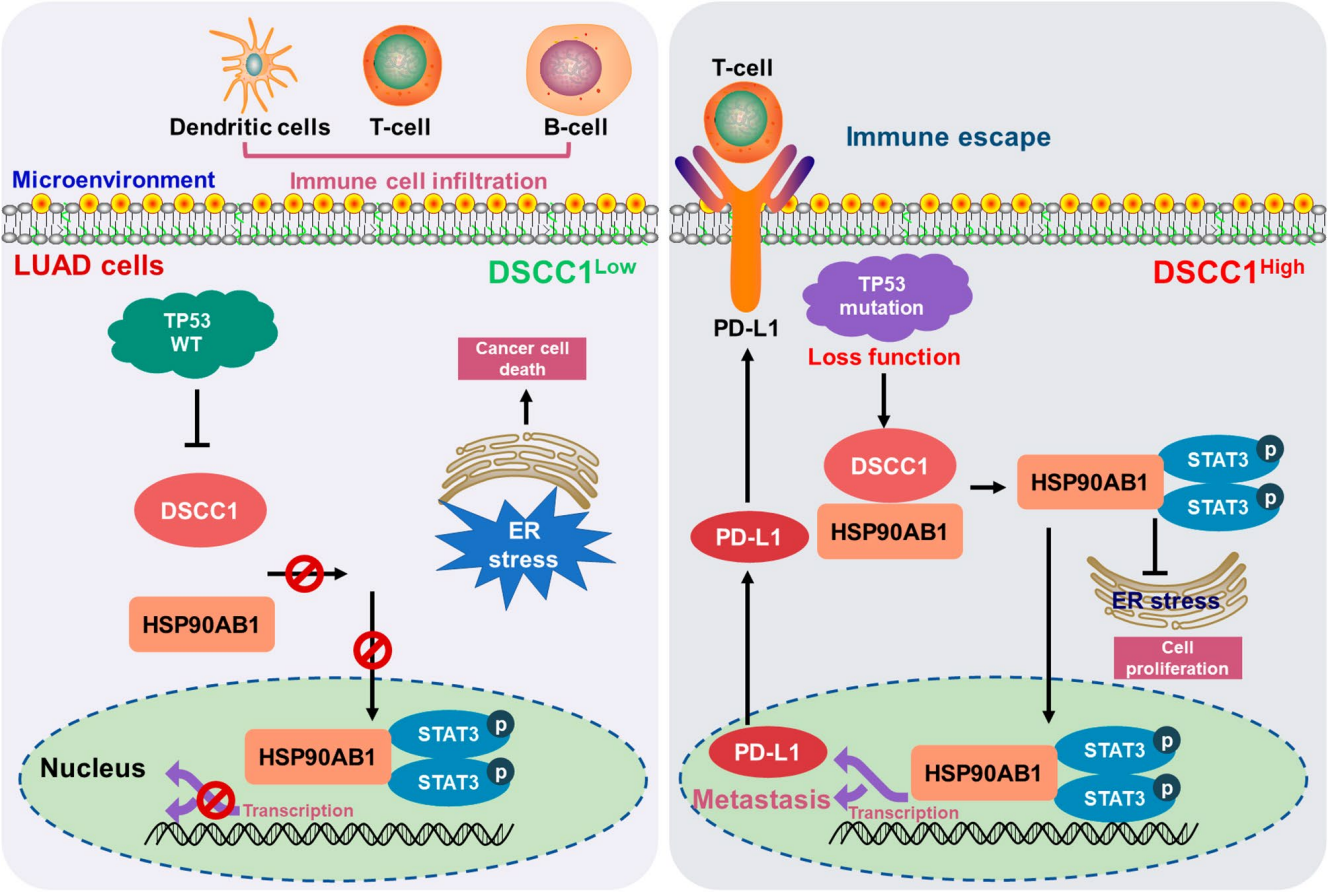
DSCC1 promotes the progression of LUAD via regulating HSP90AB1/STAT3 signaling pathway

In summary, the present study identifies a novel function of DSCC1 in LUAD progression, and HSP90AB1 is an oncogenic partner of DSCC1 in promoting LUAD progression. Furthermore, DSCC1 suppresses ER stress and promotes LUAD progress via interacting with HSP90AB1 and activating STAT3, and DSCC1 inhibits tumor immune infiltration via regulating HSP90AB1/STAT3/PD-L1 axis (Fig. 9). Meanwhile, DSCC1 might be an efficiency biomarker for predicting the clinical outcomes of PD-1/PD-L1 blockade treatment for LUAD patients. Overall, our results reveal that DSCC1 is a potentially novel therapeutic target for the treatment of LUAD and biomarker for predicting the efficiency of PD-1/PD-L1 blockade treatment.

### Electronic supplementary material

Below is the link to the electronic supplementary material.

**Additinal file 1:** Supplementary Fig. 1: the protein expression of DSCC1 was overexpressed in LUAD compared with normal lung. The immunohistochemistry data were collected from The Human Protein Atlas.

**Additinal file 2: **Supplementary Fig. 2: DSCC1 interacted protein predicted poor outcomes of LUAD patients. (A) LUAD cells were transfected with DSCC1 siRNA and control siRNA for 48 h, and RT-PCR was performed to detect the indicated mRNA level. (B) The data were obtained from TNMplot. (C) The results were collected from KM plotter. Gene-HSP90AB1, 1557910_at; Gene-PHB2, 201600_at; OS (n = 1927); FP (n = 982).

**Additinal file 3:** Supplementary Table 1: The interacting proteins of DSCC1 were identified by LC-MS/MS.

**Additinal file 4:** Supplementary Table 2: DSCC1 coexpressed genes were identified in UALCAN

**Additinal File 5:** Supplementary Table 3: Primers used in Quantitative PCR analysis

## Data Availability

The datasets used in this study are available in the TCGA repository (https://tcgadata.nci.nih.gov/tcga/) and GEO repository (http://www.ncbi.nlm.nih.gov/geo). The datasets used are available from the corresponding author on reasonable request.
