# Supplementary figures and images for: DSCC1 interacts with HSP90AB1 and promotes the progression of lung adenocarcinoma via regulating ER stress
Source: Cancer Cell Int. 2023 Sep 23;23:208. doi: 10.1186/s12935-023-03047-w (PMC10518103; doi:10.1186/s12935-023-03047-w)

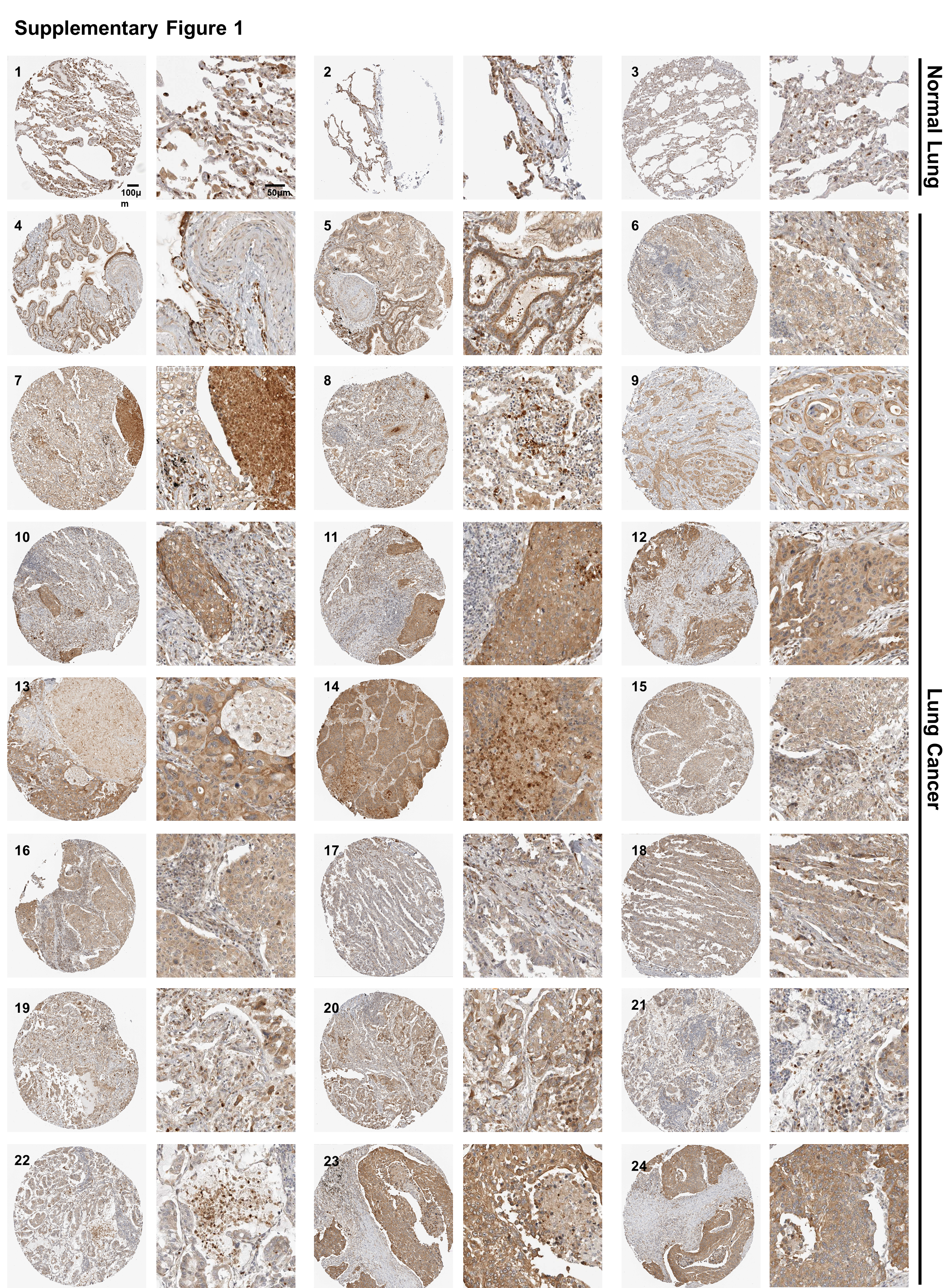

Supplement: Supplementary file 1 — Additinal file 1: Supplementary Fig. 1: the protein expression of DSCC1 was overexpressed in LUAD compared with normal lung. The immunohistochemistry data were collected from The Human Protein Atlas. [file 12935_2023_3047_MOESM1_ESM.png]

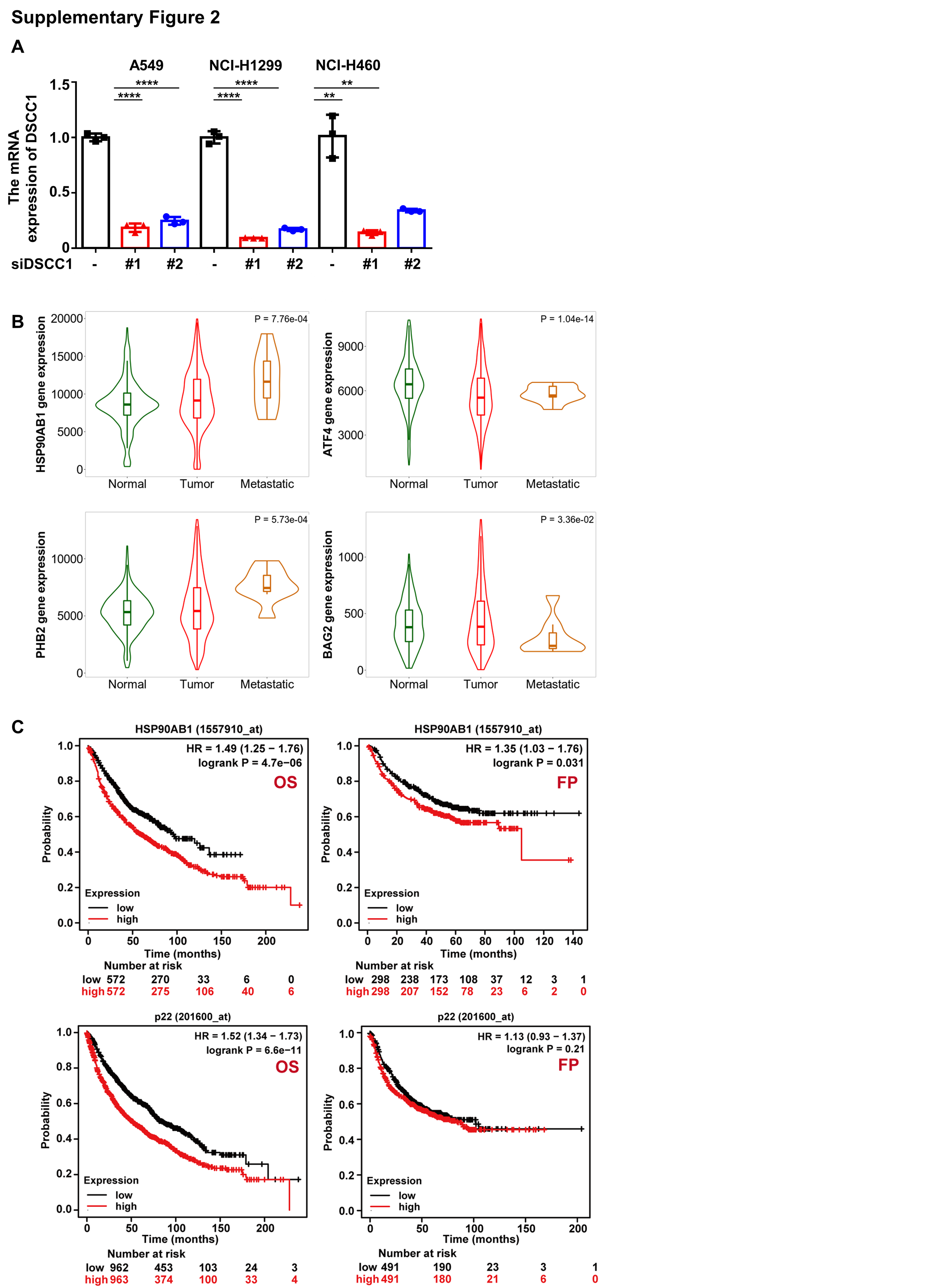

Supplement: Supplementary file 2 — Additinal file 2: Supplementary Fig. 2: DSCC1 interacted protein predicted poor outcomes of LUAD patients. (A) LUAD cells were transfected with DSCC1 siRNA and control siRNA for 48 h, and RT-PCR was performed to detect the indicated mRNA level. (B) The data were obtained from TNMplot. (C) The results were collected from KM plotter. Gene-HSP90AB1, 1557910_at; Gene-PHB2, 201600_at; OS (n = 1927); FP (n = 982). [file 12935_2023_3047_MOESM2_ESM.png]
