# Supplementary material for: DSCC1 interacts with HSP90AB1 and promotes the progression of lung adenocarcinoma via regulating ER stress
Source: Cancer Cell Int. 2023 Sep 23;23:208. doi: 10.1186/s12935-023-03047-w (PMC10518103; doi:10.1186/s12935-023-03047-w)
Supplement: Supplementary file 3 — Additinal file 3: Supplementary Table 1: The interacting proteins of DSCC1 were identified by LC-MS/MS. [file 12935_2023_3047_MOESM3_ESM.pdf]

Supplementary Table 1

The interacting proteins of DSCC1 were identified by LC-MS/MS

Majority protein IDs	Q-value	Score	MS/MS Count	Mol. weight [kDa]
K22E	0	323.31	57	65.432
TBB4B	0	131.57	24	49.83
HS71B	0	91.519	11	70.051
SBSN	0	20.44	1	60.54
K1C18	0	20.067	3	48.057
LAP2B	0	19.848	3	50.67
ATF4	0	17.04	3	38.589
HS90B	0	15.962	2	83.263
PHB2	0	15.301	2	33.296
BAG2	0	12.315	2	23.772
BAF	0	11.961	1	10.058
CMC2	0	11.857	2	74.175
NACA	0	10.727	1	23.384
XAGE1	0	10.042	1	9.0777
ATPMK	0	9.494	1	6.4575
EFTU	0	7.533	0	49.541
RLA0L	0	7.4887	1	34.364
FABP5	0	7.1158	1	15.164
MPCP	0	6.9362	1	40.094
SRSF3	0	6.7742	1	19.329
TBA1B	0	6.6338	1	50.151
DNJB6	0	6.3867	0	36.087
DNJA1	0	6.3618	1	44.868
CDSN	0.0053191	6.2385	1	51.522
CGNL1	0.0052083	6.1551	0	149.08
NRAP	0.0051546	6.1147	0	197.07
ZN227	0.0051282	6.1052	1	92.032
CYTA	0.0050761	6.084	0	11.006
ANTRL	0.0050251	6.0477	0	70.628
TOM20	0.0049261	5.9868	0	16.298
ZN320	0.004878	5.9656	2	59.326
ZN197	0.0048077	5.9296	0	118.85
CB081	0.0047619	5.8843	1	63.239
LMNA	0.0047393	5.8421	1	74.139
ZRAN1	0.004717	5.7937	1	80.966
DX39A	0.0046948	5.7611	1	49.129
DTX3L	0.0046729	5.7582	0	83.553
DCA11	0.0046512	5.7461	1	61.669
GGYF2	0.0045662	5.7089	1	150.07
FHI2A	0.0045455	5.7079	1	86.557
ST38L	0.0045249	5.6998	0	54.002
ZN831	0.0045045	5.6769	0	177.95
FA98B	0.0089686	5.6639	1	45.547
PIMT	0.0089286	5.6604	1	24.636
