# Supplementary material for: DSCC1 interacts with HSP90AB1 and promotes the progression of lung adenocarcinoma via regulating ER stress
Source: Cancer Cell Int. 2023 Sep 23;23:208. doi: 10.1186/s12935-023-03047-w (PMC10518103; doi:10.1186/s12935-023-03047-w)
Supplement: Supplementary file 4 — Additinal file 4: Supplementary Table 2: DSCC1 coexpressed genes were identified in UALCAN [file 12935_2023_3047_MOESM4_ESM.pdf]

Supplementary Table 2: DSCC1 coexpressed genes were identified in UALCAN

DSCC1 coexpresssed genes	PearsonCC
RAD54B	0.84
MTBP	0.8
WDR67	0.79
NUF2	0.78
ATAD2	0.77
SPC25	0.75
FAM64A	0.75
MCM10	0.75
RACGAP1	0.74
MCM4	0.73
DEPDC1B	0.73
PCNA	0.73
CCNE2	0.72
UBE2T	0.72
PSRC1	0.72
CHEK1	0.72
SKA1	0.72
AURKB	0.72
CCNB1	0.72
RFC5	0.72
TOP2A	0.72
ESPL1	0.71
TMPO	0.71
BUB1	0.71
VRK1	0.71
CDCA8	0.71
EPR1	0.71
PRIM1	0.71
MAD2L1	0.71
KIFC1	0.71
UNG	0.7
FBXO5	0.7
KPNA2	0.7
SUV39H2	0.7
SGOL1	0.7
NCAPG	0.7
PRC1	0.7
NCAPH	0.7
CCNA2	0.7
PBK	0.7
TPX2	0.7
GINS1	0.7
CCNB2	0.69
TTK	0.69
KIF11	0.69
ZWINT	0.69
FAM72B	0.69
KIF15	0.69
NEK2	0.69
CENPA	0.69
NUDCD1	0.69
CDCA5	0.69
CDC25A	0.69
RAD51AP1	0.69
PLK1	0.69

BUB1B	0.69
DNAJC9	0.68
CDKN3	0.68
GMNN	0.68
NUSAP1	0.68
DBF4	0.68
CDC20	0.68
DNA2	0.68
NDC80	0.68
DEPDC1	0.68
CKAP2L	0.68
FEN1	0.68
MCM6	0.68
DLGAP5	0.68
LMNB1	0.67
BIRC5	0.67
MSH2	0.67
MEST	0.67
KIF14	0.67
MELK	0.67
HELLS	0.67
TIMELESS	0.67
ARHGAP11A	0.67
HJURP	0.67
C12orf48	0.67
DCAF13	0.67
SMC2	0.67
PDSS1	0.67
BUB3	0.67
CENPO	0.67
FANCI	0.67
ZWILCH	0.67
KIF2C	0.67
KIF4A	0.66
CDC45	0.66
EZH2	0.66
KIAA1524	0.66
CDC25C	0.66
KIF20A	0.66
MTHFD2	0.66
C21orf45	0.66
RNASEH2A	0.66
CDK1	0.66
KIF18B	0.66
ASPM	0.66
ZNF367	0.66
MLF1IP	0.66
C11orf82	0.66
GSG2	0.66
TAF2	0.66
RRM2	0.65
CLSPN	0.65
NUP205	0.65
CDCA3	0.65
ORC6L	0.65
GINS2	0.65
E2F2	0.65
RAD51	0.65

FAM72D	0.65
H2AFZ	0.65
SASS6	0.65
FAM83D	0.65
CENPF	0.65
SNRPD1	0.65
SGOL2	0.65
TRAIP	0.64
SKA3	0.64
UBE2S	0.64
WDR76	0.64
RFC3	0.64
DTL	0.64
KIF23	0.64
CENPI	0.64
CENPK	0.64
CENPE	0.64
CASC5	0.64
DKC1	0.64
PRR11	0.64
POC1A	0.64
ORC1L	0.64
ESCO2	0.64
CENPH	0.64
RAD21	0.64
SPAG5	0.64
PLK4	0.64
OTUD6B	0.64
CDC6	0.64
NCAPG2	0.64
CSE1L	0.63
FAM54A	0.63
C15orf23	0.63
OIP5	0.63
CDC7	0.63
ERCC6L	0.63
ATAD5	0.63
LIN9	0.63
POP1	0.63
MCM8	0.63
TOPBP1	0.63
MCM2	0.63
RAD54L	0.63
PSMC3IP	0.63
CHAF1A	0.63
POLE2	0.63
CDT1	0.62
CDCA2	0.62
CEP76	0.62
KNTC1	0.62
CEP78	0.62
TDG	0.62
MCM7	0.62
CEP55	0.62
TROAP	0.62
TIPIN	0.62
UHRF1	0.62
WDR12	0.62

C4orf46	0.62
CENPL	0.61
MPHOSPH9	0.61
HMGB2	0.61
ANAPC7	0.61
ZC3H8	0.61
TAF5	0.61
BRCA1	0.61
FOXM1	0.61
CENPN	0.61
POLQ	0.61
FANCG	0.61
WDHD1	0.61
C1orf135	0.61
SEH1L	0.61
PGAM5	0.61
DBF4B	0.61
KIF18A	0.61
ECT2	0.61
HMMR	0.61
SEPHS1	0.6
RFC4	0.6
FAM136A	0.6
MKI67	0.6
C12orf24	0.6
NCAPD3	0.6
ASF1B	0.6
EXO1	0.6
INCENP	0.6
GTSE1	0.6
FARSB	0.6
DENR	0.6
INTS8	0.6
MASTL	0.6
NOP56	0.6
PPP1CC	0.6
ARHGAP11B	0.59
DSN1	0.59
FAM72A	0.59
DONSON	0.59
CKAP2	0.59
RRM1	0.59
CCDC99	0.59
YWHAQ	0.59
NUP37	0.59
RANBP1	0.59
RAN	0.59
C1orf112	0.59
TYMS	0.59
STMN1	0.59
CENPW	0.59
C15orf42	0.59
HSPA14	0.59
C9orf40	0.59
LOC441089	0.59
MRPL13	0.59
CDK2	0.59
STIL	0.59

PCGF6	0.59
CKAP5	0.58
CCT7	0.58
WDR43	0.58
MYBL2	0.58
GAS2L3	0.58
MTERFD1	0.58
PSMG1	0.58
KIF20B	0.58
XPO5	0.58
PNPT1	0.58
CDCA4	0.58
RECQL4	0.58
SNRPB	0.58
UBE2V2	0.58
NOL11	0.58
C3orf26	0.58
KIAA0101	0.58
ACTL6A	0.58
SLC25A32	0.58
TUBA1B	0.58
RCOR2	0.58
BRIP1	0.58
SSX2IP	0.58
PRMT5	0.57
FANCD2	0.57
PKMYT1	0.57
CHAF1B	0.57
E2F8	0.57
ENY2	0.57
C9orf140	0.57
LRPPRC	0.57
PRKDC	0.57
UTP18	0.57
SFRS9	0.57
MRPL42	0.57
GDAP1	0.57
C18orf54	0.57
CKS2	0.57
DEK	0.57
TMEM206	0.57
XRCC2	0.57
XPOT	0.57
HSPD1	0.57
PRTFDC1	0.57
RBL1	0.57
MSH6	0.57
MCM3	0.56
CHAC2	0.56
CCT4	0.56
CCNF	0.56
PPAT	0.56
C12orf73	0.56
PTDSS1	0.56
HAT1	0.56
SENP1	0.56
C16orf59	0.56
TTL	0.56

DTYMK	0.56
C9orf100	0.56
SV2A	0.56
TRIP13	0.56
AURKA	0.56
SMC6	0.55
MTHFD1	0.55
POLA2	0.55
GABPB1	0.55
LMNB2	0.55
TRMT61B	0.55
WDR75	0.55
METTL4	0.55
SSRP1	0.55
RFC2	0.55
EEF1E1	0.55
UBE2N	0.55
C6orf167	0.55
AZIN1	0.55
C5orf34	0.55
C1orf163	0.55
NCAPD2	0.55
SFRS7	0.55
EXOSC2	0.55
CBX1	0.55
SIP1	0.55
PGBD1	0.55
OLA1	0.55
HNRNPL	0.55
ALKBH2	0.55
THOC4	0.55
SPC24	0.55
MED30	0.55
CCDC58	0.55
ALS2CR4	0.54
TERF1	0.54
SAAL1	0.54
EXOSC3	0.54
PTTG1	0.54
FAM111B	0.54
UCK2	0.54
PTCD3	0.54
CHD7	0.54
SLC36A4	0.54
CACYBP	0.54
EIF2C2	0.54
RMI1	0.54
NCBP1	0.54
C2orf44	0.54
MDC1	0.54
RIOK1	0.54
DDX39	0.54
DNAH14	0.54
EIF2S1	0.54
KIAA1429	0.54
ZNF695	0.54
C10orf119	0.54
DNMT1	0.54

CENPQ	0.53
PAWR	0.53
NFKBIL2	0.53
ARMC1	0.53
CPSF3	0.53
FAM91A1	0.53
SUZ12	0.53
ILF2	0.53
E2F1	0.53
CKS1B	0.53
HDAC2	0.53
RPP30	0.53
HAUS1	0.53
CHCHD3	0.53
MND1	0.53
TUBG1	0.53
TRMT12	0.53
PAICS	0.53
TMEM48	0.53
ARL6IP6	0.53
TARS	0.53
UBA2	0.53
SLC38A1	0.53
DPY19L4	0.53
FBXO45	0.53
TIMM8A	0.53
HAUS6	0.53
RAD51C	0.53
ACYP1	0.53
GEN1	0.53
BRCA2	0.53
PIF1	0.53
POLR3F	0.53
PAK1IP1	0.53
EME1	0.53
R3HDM1	0.53
PFN2	0.53
ADSL	0.53
SHMT2	0.52
LBR	0.52
SFRS2	0.52
WASF1	0.52
HNRNPR	0.52
CCDC138	0.52
CEP152	0.52
EED	0.52
MRPL15	0.52
LOC81691	0.52
PARP2	0.52
GART	0.52
H2AFX	0.52
CCT8	0.52
HLTF	0.52
C17orf75	0.52
POLR3G	0.52
PMS1	0.52
PNO1	0.52
COPS3	0.52

C2orf69	0.52
PDXP	0.52
PRPF4	0.52
TMEM65	0.52
C13orf37	0.52
SAE1	0.52
MYBL1	0.52
MRPL30	0.52
SFRS3	0.52
NOLC1	0.52
CCT5	0.52
PPID	0.51
TOP3A	0.51
TEX10	0.51
ZNF706	0.51
RBM28	0.51
PRPF19	0.51
GLRX5	0.51
URB2	0.51
ARL5B	0.51
SNRPF	0.51
TMEM38B	0.51
C19orf48	0.51
CDK8	0.51
LOC100128191	0.51
C13orf34	0.51
RPAP3	0.51
SFRS1	0.51
TRA2B	0.51
ENOPH1	0.51
NOL10	0.51
GPN3	0.51
HES6	0.51
WHSC1	0.51
TSN	0.51
PPM1G	0.51
ZNF473	0.51
BARD1	0.51
HNRPLL	0.51
TACC3	0.51
UTP6	0.51
TPRKB	0.51
NEDD1	0.51
RAB39B	0.51
MAPKAPK5	0.51
PPIF	0.51
GMPS	0.51
BRIX1	0.5
PPIL1	0.5
TCP1	0.5
FAM161A	0.5
PHF19	0.5
SPIN4	0.5
RQCD1	0.5
MAPK8	0.5
LIG1	0.5
ZC3H15	0.5
RNASEH1	0.5

DDX55	0.5
BCCIP	0.5
MKI67IP	0.5
TCF19	0.5
SUPT16H	0.5
TAF11	0.5
CDKN2D	0.5
ABCE1	0.5
RUVBL1	0.5
FANCA	0.5
IARS	0.5
MRPL47	0.5
ACTR6	0.5
HAUS8	0.5
TUBB2B	0.5
POLE	0.5
TTF2	0.5
HNRNPC	0.5
PSAT1	0.5
CDC123	0.5
E2F6	0.5
NDUFAF4	0.5
NAA15	0.5
HSPE1	0.5
HYLS1	0.5
RIC8B	0.5
WDYHV1	0.5
CCDC59	0.5
SLBP	0.5
STOML2	0.5
NAA25	0.5
LYAR	0.5
SNRPC	0.5
ACLY	0.5
SKP2	0.5
KDM1A	0.5
VDAC3	0.5
VTA1	0.5
STIP1	0.5
C8orf33	0.5
MTFR1	0.5
PA2G4	0.5
SCLT1	0.49
GPR19	0.49
PPIL5	0.49
POLD3	0.49
TBP	0.49
COPS5	0.49
HSF2	0.49
FANCC	0.49
MNAT1	0.49
MDH1	0.49
THOP1	0.49
RNF138	0.49
USP37	0.49
MRPL3	0.49
GTPBP4	0.49
KHSRP	0.49

TIMM44	0.49
E2F3	0.49
SMC3	0.49
PRPSAP2	0.49
C11orf84	0.49
OBFC2B	0.49
AIMP2	0.49
ESF1	0.49
DDX18	0.49
PSMD12	0.49
BRI3BP	0.49
DTD1	0.49
RFWD3	0.49
PRMT3	0.49
FASTKD1	0.49
ACBD7	0.49
DIAPH3	0.49
CCNJ	0.49
DDX1	0.49
MLLT11	0.49
EXOC5	0.49
HSPA4L	0.49
PAXIP1	0.49
C17orf96	0.49
RBM17	0.49
TRMT6	0.49
PHGDH	0.49
SENP3	0.49
SNRNP48	0.49
POLR3A	0.49
TIGD3	0.49
RNF34	0.49
VBP1	0.49
EXOSC8	0.49
UBE2C	0.49
KPNB1	0.49
C17orf80	0.49
TFAM	0.49
GLMN	0.49
GATC	0.49
LIN54	0.49
KATNA1	0.49
C12orf29	0.49
MTPAP	0.49
TMEM194A	0.49
PSME3	0.49
EPT1	0.48
RG9MTD1	0.48
NUP85	0.48
CCT3	0.48
IFRD1	0.48
POLR2D	0.48
GINS4	0.48
PRMT1	0.48
NUP153	0.48
STRAP	0.48
ZNF286A	0.48
CHORDC1	0.48

C13orf27	0.48
MPP6	0.48
NAA50	0.48
NLN	0.48
XPO1	0.48
MRPL11	0.48
H2AFV	0.48
MTIF2	0.48
MAP6D1	0.48
TBPL1	0.48
MRTO4	0.48
SSB	0.48
MARS2	0.48
YBX2	0.48
ZRANB3	0.48
MRPL19	0.48
AHCY	0.48
DUT	0.48
RBMX	0.48
PHF20L1	0.48
G2E3	0.48
DHFR	0.48
YWHAZ	0.48
LOC221710	0.48
ELAVL1	0.48
MTCH2	0.48
BLM	0.48
TUBB	0.48
L2HGDH	0.48
SET	0.48
CBS	0.48
PWP1	0.48
C20orf72	0.48
DARS2	0.48
HPRT1	0.48
CENPM	0.48
NUP35	0.48
C2orf3	0.48
REEP2	0.48
LTV1	0.48
DHX33	0.48
PRKRIR	0.48
FAM49B	0.48
GRPEL2	0.47
TOMM70A	0.47
CCDC21	0.47
KHK	0.47
LRRC40	0.47
KRR1	0.47
EIF2S2	0.47
GTF3C3	0.47
HMGN4	0.47
OPA1	0.47
YRDC	0.47
MRPS16	0.47
SMNDC1	0.47
KIAA0406	0.47
ZNF623	0.47

ERH	0.47
MPP2	0.47
UTP23	0.47
PUF60	0.47
WDR62	0.47
ZC3HC1	0.47
CENPP	0.47
NFXL1	0.47
EIF4A3	0.47
ANAPC5	0.47
GTF2H3	0.47
CASP8AP2	0.47
CECR5	0.47
ESRP1	0.47
POLR2K	0.47
NRCAM	0.47
PIGW	0.47
TATDN1	0.47
USP1	0.47
IMMT	0.47
ANAPC1	0.47
NAE1	0.47
SUPV3L1	0.47
ATP13A3	0.47
C6orf150	0.47
ZNF639	0.47
PSMD14	0.47
RPF2	0.47
SAP30	0.47
CUL2	0.47
HMGXB4	0.46
NCL	0.46
RIMKLA	0.46
PUS7	0.46
DCK	0.46
RDBP	0.46
CYC1	0.46
ZC3HAV1L	0.46
TMSB15A	0.46
MYO19	0.46
FAM158A	0.46
CCDC34	0.46
C17orf53	0.46
RAE1	0.46
CSTF3	0.46
COMMD2	0.46
PL-5283	0.46
SUDS3	0.46
BOP1	0.46
C6orf182	0.46
APEX1	0.46
METTL2A	0.46
MTL5	0.46
KLHL23	0.46
CCDC86	0.46
C1orf109	0.46
RNF157	0.46
RPE	0.46

MFN1	0.46
ACP1	0.46
ECE2	0.46
PDRG1	0.46
UBR5	0.46
SLC25A13	0.46
TRIM59	0.46
CYCS	0.46
DARS	0.46
PSIP1	0.46
TBRG4	0.46
CEP97	0.46
CCDC43	0.46
PSMD11	0.46
PARG	0.46
RPAP2	0.46
CPSF2	0.46
NRM	0.46
MAPRE1	0.46
CDH24	0.46
PM20D2	0.46
PHF6	0.46
PNMA1	0.45
NUDT1	0.45
MTHFD1L	0.45
DDX21	0.45
SNRPB2	0.45
GTF2E1	0.45
EFTUD2	0.45
LSM5	0.45
NUP160	0.45
FIGNL1	0.45
ASNS	0.45
TSR1	0.45
CDC5L	0.45
DHX36	0.45
ANKRD13B	0.45
NME1	0.45
SPATS2	0.45
NOP58	0.45
CHEK2	0.45
NT5C3	0.45
ABHD10	0.45
PTBP1	0.45
NSMCE2	0.45
FBXO30	0.45
NIF3L1	0.45
NUP43	0.45
C20orf27	0.45
TOMM5	0.45
PPP1R8	0.45
POLA1	0.45
TRIM28	0.45
SLC7A1	0.45
COQ3	0.45
UBR7	0.45
CHRAC1	0.45
TMEM106C	0.45

FARSA	0.45
LRRCC1	0.45
BOLA3	0.45
NARS2	0.45
PTGES3	0.45
FAM24B	0.45
RIF1	0.45
PREB	0.45
C6orf129	0.45
RAD18	0.45
UMPS	0.45
IQGAP3	0.45
ACTR3B	0.45
ILF3	0.45
NONO	0.44
MYL6B	0.44
DSP	0.44
NKRF	0.44
MRPL35	0.44
C17orf42	0.44
COQ5	0.44
MEMO1	0.44
SBNO1	0.44
C12orf34	0.44
MRE11A	0.44
XRCC3	0.44
TADA2A	0.44
FAM60A	0.44
C4orf21	0.44
HNRNPM	0.44
C2orf49	0.44
MAGOHB	0.44
QSOX2	0.44
TGS1	0.44
UPF3B	0.44
RDM1	0.44
ITGB3BP	0.44
SNRPA1	0.44
FBL	0.44
KHDRBS1	0.44
NOC3L	0.44
DDX10	0.44
RPA3	0.44
SNHG1	0.44
YEATS2	0.44
CAD	0.44
TACO1	0.44
METTL10	0.44
MRPS30	0.44
KIF24	0.44
VPS33A	0.44
NUP155	0.44
AURKAPS1	0.44
HMGN2	0.44
GNAZ	0.44
TUBA1C	0.44
LSM2	0.44
XRCC5	0.44

C2orf47	0.44
SRPK2	0.44
FGD1	0.44
PDK1	0.44
SART3	0.44
UBXN2A	0.44
MED27	0.44
DIABLO	0.44
CBX3	0.44
EIF2B1	0.44
GTPBP8	0.44
C20orf7	0.44
STRBP	0.44
CEBPZ	0.44
HNRNPH3	0.44
PDCD2	0.44
SHCBP1	0.44
ZNF775	0.43
CDKAL1	0.43
TCEB1	0.43
NBN	0.43
METTL8	0.43
MARK1	0.43
ATP5B	0.43
C18orf56	0.43
PSME4	0.43
PARP1	0.43
CENPV	0.43
HBS1L	0.43
NUDT15	0.43
SRPK1	0.43
ATP6V1C1	0.43
LRP12	0.43
CIRH1A	0.43
FANCL	0.43
ZBTB39	0.43
AHSA1	0.43
HNRNPF	0.43
PSMB5	0.43
MARS	0.43
DERL1	0.43
KIF2A	0.43
WBP11	0.43
CDC27	0.43
ATP6V1G2	0.43
AGPAT5	0.43
PTPN11	0.43
SMN2	0.43
C10orf88	0.43
HAUS2	0.43
MCM5	0.43
DPM1	0.43
SYNJ2	0.43
PRPF40A	0.43
WDR5	0.43
UCHL5	0.43
LOC730101	0.43
KPNA4	0.43

HNRNPA2B1	0.43
CRMP1	0.43
NLE1	0.43
HSP90AA1	0.43
HSPA13	0.43
SLC25A3	0.43
RSRC1	0.43
LUC7L2	0.43
ABCF2	0.43
YBX1	0.43
BLMH	0.43
USP39	0.43
MTA2	0.43
RPL7L1	0.43
MRPL48	0.43
RRP1B	0.43
PUS7L	0.43
DHX9	0.43
STC2	0.43
SKA2	0.43
SFRS13B	0.43
IPO5	0.43
FAM104A	0.43
AP1AR	0.43
CRY1	0.43
POLR2H	0.43
ACN9	0.43
C16orf61	0.43
SNRNP40	0.43
DCUN1D5	0.42
RUVBL2	0.42
NOL7	0.42
KIAA1586	0.42
SEC61A2	0.42
SFPQ	0.42
PTMA	0.42
BAG2	0.42
ANP32B	0.42
BBS7	0.42
METTL2B	0.42
POLE3	0.42
ZNF620	0.42
CLPB	0.42
C8orf37	0.42
CHUK	0.42
MAZ	0.42
C12orf43	0.42
FYTTD1	0.42
C8orf76	0.42
CISD1	0.42
ZNHIT3	0.42
TMEM199	0.42
TIAL1	0.42
E2F7	0.42
POLD1	0.42
CTDSPL2	0.42
NAA35	0.42
NUP210	0.42

SUMO2	0.42
ORC5L	0.42
ANLN	0.42
IPO4	0.42
PPIH	0.42
PCMT1	0.42
AFG3L2	0.42
SNRPE	0.42
DNAJC6	0.42
SUV39H1	0.42
NMD3	0.42
CCRN4L	0.42
RCC2	0.42
EXOSC9	0.42
WDR4	0.42
ZFR	0.42
NDUFA9	0.42
DDX11	0.42
NASP	0.42
SERF1A	0.42
SMAD9	0.42
NGDN	0.42
REPS1	0.42
SF3B14	0.42
ZNF643	0.42
ZUFSP	0.42
TRMT5	0.42
SYT1	0.42
CSTF2	0.42
ARL6IP1	0.42
FOXK2	0.42
USP14	0.42
ZNF232	0.42
GMFB	0.42
C18orf19	0.42
SNHG6	0.42
GINS3	0.42
DHX37	0.42
DDX12	0.42
FAM98B	0.42
C10orf2	0.42
C14orf33	0.42
C16orf75	0.42
UCHL1	0.42
SMC4	0.42
MRPS22	0.42
HOMER1	0.42
MTRF1L	0.42
IPO9	0.41
C11orf73	0.41
SOCS4	0.41
CBX2	0.41
DDX50	0.41
C1orf96	0.41
EIF4E	0.41
LCLAT1	0.41
NAA38	0.41
RBBP7	0.41

TOMM40	0.41
SFXN4	0.41
MEX3B	0.41
LSG1	0.41
PFKM	0.41
TMEM201	0.41
FADS1	0.41
DNMT3A	0.41
POT1	0.41
GPRIN1	0.41
EIF2B3	0.41
YY1	0.41
TAF1A	0.41
PARL	0.41
MAD2L2	0.41
ERAL1	0.41
SLMO1	0.41
SF3A3	0.41
ATP6V0A2	0.41
SNW1	0.41
TCERG1	0.41
EBNA1BP2	0.41
ZNF22	0.41
CSNK2A1P	0.41
SFRS13A	0.41
MOBKL3	0.41
PPT2	0.41
SLC5A6	0.41
SETD8	0.41
ASAP1	0.41
REPIN1	0.41
CBLL1	0.41
METTL5	0.41
NPM3	0.41
PRPSAP1	0.41
FAM83F	0.41
NDUFS1	0.41
TPI1	0.41
DLEU2	0.41
GGH	0.41
HNRNPA3P1	0.41
FSD1L	0.41
DCAF17	0.41
RPIA	0.41
SLC25A33	0.41
LIG3	0.41
NEIL3	0.41
YDJC	0.41
EIF5AL1	0.41
MSI2	0.41
QSER1	0.41
PATL1	0.41
MRPL45	0.41
TET3	0.41
DCLRE1A	0.41
NRAS	0.41
TK1	0.41
EIF4A1	0.41

PDCD11	0.41
ZBTB12	0.4
NEFH	0.4
POLD2	0.4
ZNF146	0.4
PCNP	0.4
CCNB1IP1	0.4
MRPL12	0.4
CTPS	0.4
DPH2	0.4
TMEM198	0.4
PAIP1	0.4
LSM12	0.4
RBM38	0.4
PGAM1	0.4
PHB	0.4
GPI	0.4
TMTC3	0.4
C11orf30	0.4
PTS	0.4
KLHL7	0.4
DUS4L	0.4
NARS	0.4
TMEM209	0.4
TRIAP1	0.4
DRG1	0.4
PSMA2	0.4
CDK5R1	0.4
QTRTD1	0.4
SERBP1	0.4
ZNF280C	0.4
PXMP2	0.4
ABT1	0.4
C8orf59	0.4
GEMIN8P4	0.4
PFDN4	0.4
CPT1C	0.4
C14orf106	0.4
ZW10	0.4
UBE2M	0.4
RBBP4	0.4
C14orf135	0.4
SMCR7L	0.4
ITGB1BP1	0.4
NUP54	0.4
MRPL37	0.4
FANCM	0.4
ABCF1	0.4
ARG2	0.4
IMPA1	0.4
CSNK2A1	0.4
C6orf153	0.4
RP9P	0.4
ZNF326	0.4
NUP62	0.4
C8orf39	0.4
SPAST	0.4
DLL1	0.4

PRPS1	0.4
C3orf37	0.4
ORC2L	0.4
HNRNPA3	0.4
CLNS1A	0.4
AP3M2	0.4
ZNF239	0.4
IMPAD1	0.4
METAP2	0.4
MRPL51	0.4
SYCE2	0.4
ZNF519	0.4
TRIM37	0.4
TMED10P1	0.4
RNGTT	0.4
DAZAP1	0.4
IGF2BP3	0.4
BMS1	0.4
CPSF6	0.4
LZIC	0.4
GFM1	0.4
RPA1	0.4
PDCL3	0.39
C1QBP	0.39
TOMM22	0.39
RDH11	0.39
TDP1	0.39
EIF3J	0.39
ARMC8	0.39
SMC1A	0.39
HDGFRP3	0.39
UNK	0.39
SAMD1	0.39
CNOT10	0.39
FXR1	0.39
FAM119A	0.39
GLRX3	0.39
COPS8	0.39
CEBPG	0.39
SLC25A19	0.39
DLAT	0.39
G3BP1	0.39
POP7	0.39
C7orf11	0.39
SFXN1	0.39
WDR53	0.39
TSSC1	0.39
SMARCB1	0.39
FAM92A1	0.39
GCH1	0.39
SAPS3	0.39
TSGA14	0.39
SIGMAR1	0.39
PPP2R5D	0.39
WSB2	0.39
PRKRA	0.39
MRPS9	0.39
RBM14	0.39

SYNCRIP	0.39
KIAA1549	0.39
SNRNP27	0.39
AACS	0.39
VRK2	0.39
DNAJB11	0.39
C12orf41	0.39
POLR1B	0.39
WDR74	0.39
CCDC110	0.39
RNF2	0.39
C14orf80	0.39
ROBO1	0.39
LARP1B	0.39
RNF219	0.39
H2AFY	0.39
AK3L1	0.39
CBFA2T2	0.39
VPS29	0.39
BUD13	0.39
DVL2	0.39
YWHAE	0.39
PDCD10	0.39
NXPH4	0.39
THAP1	0.39
LOC388796	0.39
C18orf55	0.39
TUBGCP4	0.39
UBE2CBP	0.39
HOOK1	0.39
NFYB	0.39
GTF2H4	0.39
ISY1	0.39
PSMA7	0.39
GPN1	0.39
CCDC15	0.39
MRPL21	0.39
GKAP1	0.39
SSBP1	0.39
ZNF572	0.39
PAPOLA	0.39
LRRC42	0.39
TMEM185B	0.39
PSMD2	0.39
METAP1	0.39
MRPS10	0.39
MTDH	0.39
UBE2MP1	0.39
NETO2	0.39
DNAJA1	0.39
CISD2	0.39
TMSB15B	0.39
SLC7A5	0.39
HSF1	0.39
CCDC150	0.39
FANCE	0.39
CRNKL1	0.38
HS2ST1	0.38

FKBP4	0.38
CENPJ	0.38
TBCCD1	0.38
RB1CC1	0.38
HAUS7	0.38
HAUS3	0.38
SENP5	0.38
RBM12	0.38
DHX57	0.38
CCDC112	0.38
C14orf145	0.38
DNM1	0.38
CCDC18	0.38
KIAA0586	0.38
UBE2K	0.38
HNRNPD	0.38
GCN1L1	0.38
SCO1	0.38
NOM1	0.38
FAF1	0.38
PDCD2L	0.38
ILKAP	0.38
ATP5C1	0.38
UTP11L	0.38
CCAR1	0.38
LUZP6	0.38
RAB6A	0.38
INTS2	0.38
RND2	0.38
WRNIP1	0.38
WDR3	0.38
RECQL	0.38
RPP40	0.38
DCLRE1B	0.38
EHBP1	0.38
DCUN1D1	0.38
DCTPP1	0.38
CPSF4	0.38
PPP2R5E	0.38
AGMAT	0.38
DHX15	0.38
TRUB1	0.38
RNF8	0.38
DDX47	0.38
NOP2	0.38
NEU3	0.38
ZFP30	0.38
C8orf38	0.38
POFUT1	0.38
C15orf41	0.38
HNRNPK	0.38
TARDBP	0.38
CCDC85C	0.38
ACTR3	0.38
LYPLA1	0.38
CS	0.38
GFM2	0.38
MAPK6	0.38

GARS	0.38
PTMS	0.38
C4orf43	0.38
TCOF1	0.38
SLC16A1	0.38
NKIRAS2	0.38
LOC144438	0.38
RBM12B	0.38
ZCCHC8	0.38
GPSM2	0.38
ING3	0.38
STAU2	0.38
VPS25	0.38
MKKS	0.38
PDHX	0.38
MTA1	0.38
RAB10	0.38
C14orf156	0.38
LRRC58	0.38
E2F5	0.38
IGFBPL1	0.38
NDUFB9	0.38
CCDC77	0.38
SNRPG	0.38
EIF4EBP1	0.38
PPM1D	0.38
C17orf79	0.38
UHRF1BP1L	0.38
SOCS7	0.38
DBR1	0.38
SUMO1	0.37
AGPS	0.37
ERCC8	0.37
ACPL2	0.37
BANF1	0.37
INTS7	0.37
NDUFA12	0.37
SRM	0.37
KLC2	0.37
KDM2B	0.37
SMARCD1	0.37
C17orf71	0.37
GTF3C2	0.37
BTBD3	0.37
SAP130	0.37
NUS1	0.37
COG5	0.37
PTBP2	0.37
CAMK2N2	0.37
POLR2G	0.37
SMCHD1	0.37
GNA13	0.37
U2AF2	0.37
DYNLL1	0.37
KIF5B	0.37
COIL	0.37
RRP9	0.37
SNAP25	0.37

HTRA2	0.37
TRIB3	0.37
GSTCD	0.37
LOC401431	0.37
RINT1	0.37
MINPP1	0.37
KIAA1731	0.37
MAGOH	0.37
HIRA	0.37
SUPT3H	0.37
RHEBL1	0.37
ZNF259	0.37
UHRF1BP1	0.37
CBX5	0.37
SMARCE1	0.37
UBXN7	0.37
ZNF711	0.37
GTF3C5	0.37
TMEM68	0.37
LARP4	0.37
LOC341056	0.37
NUP188	0.37
MRPL39	0.37
MRPL2	0.37
NEDD8	0.37
HAUS5	0.37
DLD	0.37
PCCB	0.37
EIF3H	0.37
RAP2A	0.37
C2orf15	0.37
TLK2	0.37
C16orf88	0.37
U2AF1	0.37
YME1L1	0.37
LRRC61	0.37
C12orf32	0.37
BAG4	0.37
AGBL5	0.37
SMPD4	0.37
DYM	0.37
BTF3L4	0.37
TUBB2A	0.37
VDAC2	0.37
POLR3B	0.37
ATP5G3	0.37
STAG1	0.37
PSPH	0.37
AKIRIN2	0.37
ZNF670	0.37
PUS1	0.37
MRPS7	0.37
C3orf17	0.37
ATAD3A	0.37
RCCD1	0.37
TIMM9	0.37
CNIH2	0.37
APTX	0.37

YES1	0.37
ZNF74	0.37
MPHOSPH10	0.37
SR140	0.37
NOP14	0.37
DNM1L	0.37
GMCL1	0.37
ZCCHC7	0.37
LSM11	0.37
FGFR1OP	0.37
AAGAB	0.37
USP42	0.36
ASNSD1	0.36
C9orf30	0.36
PPP4R2	0.36
ORC4L	0.36
SCYL2	0.36
ANKIB1	0.36
SCRIB	0.36
C10orf18	0.36
CRKL	0.36
CCDC88A	0.36
C6orf162	0.36
LRRC8D	0.36
CTSL2	0.36
C7orf49	0.36
TGIF2	0.36
EIF2C3	0.36
SRRT	0.36
EXTL2	0.36
C16orf87	0.36
MICAL3	0.36
PGAM4	0.36
RPF1	0.36
LOC642846	0.36
GNL3	0.36
HCFC1	0.36
POLR1A	0.36
PSIMCT-1	0.36
SMARCC1	0.36
MCRS1	0.36
FAM133B	0.36
ZBTB2	0.36
ADO	0.36
HCN3	0.36
RANGAP1	0.36
DHX35	0.36
DNMT3B	0.36
MDH2	0.36
NUFIP1	0.36
RBM45	0.36
TUSC3	0.36
COPG2	0.36
C5orf22	0.36
B4GALNT4	0.36
DCAF16	0.36
PHF5A	0.36
DVL3	0.36

RTTN	0.36
MAP1B	0.36
ATIC	0.36
ZNF511	0.36
ZNF3	0.36
CFL2	0.36
ADNP	0.36
N4BP2	0.36
KIAA0196	0.36
TMED2	0.36
PRDX3	0.36
MED17	0.36
HSD17B10	0.36
MRPL52	0.36
GANAB	0.36
SCFD1	0.36
TMEM70	0.36
PPP3R1	0.36
BAT2	0.36
MYEF2	0.36
KCNH2	0.36
CDH2	0.36
TFRC	0.36
THOC3	0.36
MTMR2	0.36
MRPL1	0.36
GSK3B	0.36
MTF2	0.36
UBE2O	0.36
MAFG	0.36
AKT3	0.36
B3GNT5	0.36
YTHDF3	0.36
ZNF765	0.36
PTCD1	0.36
UBE2G1	0.36
RHEB	0.36
ABI2	0.36
USP18	0.36
SF3B2	0.36
PSMD9	0.36
NHLRC2	0.36
MEX3A	0.36
C20orf20	0.36
TWF1	0.36
CASC3	0.36
MRPS33	0.36
SGTB	0.36
GIT1	0.36
ACAT2	0.36
LDLRAD3	0.36
MED28	0.36
PHF10	0.36
FAM199X	0.35
HMBS	0.35
FAM20B	0.35
C12orf11	0.35
UFD1L	0.35

PPHLN1	0.35
NCBP2	0.35
TMX1	0.35
WAC	0.35
PITPNB	0.35
RBM15	0.35
PSMD3	0.35
UCHL3	0.35
GATSL1	0.35
PCBP2	0.35
BEND3	0.35
PSMA3	0.35
ZYG11A	0.35
LOC728819	0.35
THOC1	0.35
DNAJC2	0.35
TTPAL	0.35
B3GALNT2	0.35
KIAA0020	0.35
SMS	0.35
ZSCAN21	0.35
C14orf104	0.35
DAXX	0.35
GNAI3	0.35
MOBKL1B	0.35
CNKSR3	0.35
5-Mar	0.35
ZFP64	0.35
TMEFF1	0.35
KIF3C	0.35
GSS	0.35
ACOT7	0.35
LIN52	0.35
STK3	0.35
TXNL4A	0.35
UBE2L3	0.35
ZNF664	0.35
SNF8	0.35
ZNF143	0.35
C1orf124	0.35
CEP135	0.35
KIAA1958	0.35
NANP	0.35
KPNA1	0.35
PIAS2	0.35
HIC2	0.35
KIN	0.35
EIF5A	0.35
SLMO2	0.35
NLK	0.35
FECH	0.35
UBAP2	0.35
CCT6A	0.35
SPATA5L1	0.35
YARS2	0.35
RAD51L3	0.35
IDH3B	0.35
MIB1	0.35

ZNF48	0.35
KIAA1715	0.35
CARS	0.35
PPP1R12A	0.35
ZNF530	0.35
FIGN	0.35
TEAD4	0.35
TCEA1	0.35
ADNP2	0.35
NUDT21	0.35
UBE2E3	0.35
TSEN15	0.35
FAM169A	0.35
ERCC3	0.35
SPIN1	0.35
ZNF696	0.35
MRPL44	0.35
TAF6	0.35
PLCB1	0.35
SENP2	0.35
TCF3	0.35
LMBR1	0.35
XRN2	0.35
CEP72	0.35
MAPK8IP2	0.35
SGTA	0.35
GRK4	0.35
MCM3APAS	0.35
RBM23	0.35
AGK	0.35
TXNDC9	0.35
CSRP2	0.35
SDAD1	0.35
FAM76B	0.35
FOXJ3	0.35
HSP90AB1	0.35
ADAM17	0.35
KLHL11	0.35
LSM14A	0.35
CDCA7L	0.35
MIAT	0.35
PSMA4	0.35
JARID2	0.35
C19orf57	0.35
STRN	0.35
CREB1	0.34
KTN1	0.34
LAPTM4B	0.34
STARD7	0.34
IPMK	0.34
SMU1	0.34
PAK2	0.34
PIGX	0.34
CNBP	0.34
LOC727896	0.34
COMMD8	0.34
SLC25A40	0.34
YWHAG	0.34

TMEM97	0.34
RAC3	0.34
AASDHPPT	0.34
ZNF252	0.34
RNF26	0.34
ZDHHC23	0.34
HMGA1	0.34
TLK1	0.34
SIX2	0.34
TBC1D16	0.34
EIF2AK2	0.34
FKBP3	0.34
HPDL	0.34
CDC23	0.34
MIS12	0.34
ALMS1	0.34
PNP	0.34
C15orf61	0.34
RIPK2	0.34
NAA20	0.34
HEATR1	0.34
PPAN	0.34
LOC728640	0.34
MRPS5	0.34
MMD	0.34
ANKRD27	0.34
CASP2	0.34
TH1L	0.34
SEC11C	0.34
C14orf21	0.34
COPS6	0.34
TIPRL	0.34
PACRGL	0.34
MORF4L2	0.34
PRKAB2	0.34
ISG20L2	0.34
MED6	0.34
TRERF1	0.34
DOT1L	0.34
UBE3C	0.34
C3orf67	0.34
MTERF	0.34
PAQR3	0.34
ANKRD26	0.34
RAD9B	0.34
PTGES2	0.34
PSPC1	0.34
TNPO2	0.34
DEPDC4	0.34
TFDP2	0.34
ZNF318	0.34
EXOSC10	0.34
DNAJC7	0.34
DDX52	0.34
C17orf58	0.34
C1orf74	0.34
PHF14	0.34
MMP12	0.34

YAF2	0.34
NAIF1	0.34
SP3	0.34
WRAP53	0.34
VAPB	0.34
PSMB2	0.34
ZNF124	0.34
PKNOX1	0.34
RAPGEF4	0.34
TMEM194B	0.34
GNPNAT1	0.34
LOC729020	0.34
LCORL	0.34
C7orf44	0.34
PDCD5	0.34
LSM4	0.34
NRF1	0.34
JRK	0.34
MGC57346	0.34
GCLC	0.34
TNPO1	0.34
ZNF496	0.34
KIF21A	0.34
PRKAA2	0.34
PPME1	0.34
WDR35	0.34
CSTF1	0.34
ZNF131	0.34
DNAJC11	0.34
ALS2	0.34
ESCO1	0.34
EIF3B	0.34
MRPS17	0.34
RALA	0.34
PSMC3	0.34
CHFR	0.33
AKIRIN1	0.33
ZNF16	0.33
IPPK	0.33
PPP1CB	0.33
MTX2	0.33
EHMT2	0.33
TTC5	0.33
ATG4D	0.33
C6orf125	0.33
CTNNAL1	0.33
PEMT	0.33
STRN4	0.33
PHTF2	0.33
ATP2B1	0.33
NOC4L	0.33
C10orf12	0.33
GLA	0.33
PAFAH1B3	0.33
PGAP1	0.33
WDR77	0.33
ATF2	0.33
FAM175B	0.33

MSL3L2	0.33
TAF3	0.33
SNRNP200	0.33
GTF2A1	0.33
PRDM4	0.33
FBXO33	0.33
MAP2K6	0.33
ITPRIPL1	0.33
NIP7	0.33
LSM6	0.33
BCL2L11	0.33
C1orf59	0.33
INTS4	0.33
ZNF525	0.33
ZNF93	0.33
PPP2R3C	0.33
NUDT5	0.33
TAF9	0.33
PPP5C	0.33
DYNC1LI1	0.33
MPHOSPH6	0.33
ZNF544	0.33
APOO	0.33
CEP192	0.33
KBTBD2	0.33
ZNF697	0.33
HMGB1	0.33
CTNNBL1	0.33
SNRPA	0.33
RNFT2	0.33
DFFA	0.33
TNPO3	0.33
LRWD1	0.33
GDI2	0.33
UTP20	0.33
NSMCE4A	0.33
APOBEC3B	0.33
LOC441046	0.33
AMMECR1	0.33
CSPP1	0.33
ZKSCAN1	0.33
FASTKD2	0.33
ZNF507	0.33
RPL39L	0.33
TBCB	0.33
TAF1B	0.33
ELOVL7	0.33
ZNF92	0.33
BRAP	0.33
GPR137C	0.33
C6orf136	0.33
PSMD7	0.33
FAM55C	0.33
KIAA0090	0.33
IMMP1L	0.33
NME1-NME2	0.33
PSTK	0.33
SLC25A39	0.33

MAGEF1	0.33
FH	0.33
ASH2L	0.33
STAMBP	0.33
SARNP	0.33
TFCP2	0.33
TUBGCP3	0.33
FUBP3	0.33
PSMB6	0.33
FAR2	0.33
NT5DC3	0.33
NDUFB5	0.33
TIMM10	0.33
CDC73	0.33
ZNF280B	0.33
PSMD1	0.33
NOL9	0.33
RBM8A	0.33
C9orf80	0.33
COX7A2L	0.33
IMP4	0.33
NUP88	0.33
MRPL9	0.33
C2orf43	0.33
C14orf118	0.33
UBTF	0.33
RRP15	0.33
HSPA4	0.33
DDX46	0.33
ATF4	0.33
C6orf115	0.33
UBA6	0.33
XYLB	0.33
DNAJC10	0.33
DGUOK	0.33
GPS1	0.33
PSMB7	0.33
MARCKSL1	0.33
GRP	0.33
DCAF7	0.33
ZNF195	0.33
OSGIN2	0.33
MED1	0.33
ISCA1P1	0.33
BYSL	0.33
MUDENG	0.33
LSM3	0.33
THAP5	0.33
TRPM2	0.33
EIF5B	0.33
ERLIN1	0.33
MINA	0.33
EXOSC4	0.33
USP5	0.33
PHLPP2	0.33
FKBPL	0.33
NOC2L	0.33
CCDC123	0.33

TXNL1	0.33
DGKG	0.33
ZFP91	0.33
SLC30A6	0.33
DR1	0.33
DUS3L	0.32
SNAPC1	0.32
RP9	0.32
KLHDC3	0.32
HDAC1	0.32
METTL11A	0.32
GTF2F2	0.32
KIF3B	0.32
SCLY	0.32
DIP2B	0.32
6-Mar	0.32
SLC4A1AP	0.32
HDGF	0.32
PRR3	0.32
ARHGAP39	0.32
DHX16	0.32
3-Sep	0.32
GLOD4	0.32
KIAA1841	0.32
TTC27	0.32
CCNT1	0.32
NUP107	0.32
DNTTIP2	0.32
ASXL1	0.32
SOX2	0.32
YIF1B	0.32
C19orf40	0.32
ARID3A	0.32
CPSF7	0.32
SOX12	0.32
RNASEN	0.32
NAF1	0.32
SNRPD3	0.32
LOC222699	0.32
C3orf21	0.32
TUBD1	0.32
SNX16	0.32
AGTPBP1	0.32
PHB2	0.32
MYC	0.32
PES1	0.32
NUTF2	0.32
C20orf11	0.32
CIB2	0.32
GLRX2	0.32
PLAA	0.32
CDV3	0.32
CDC34	0.32
LARS2	0.32
TIGD5	0.32
IFT52	0.32
MAPK12	0.32
CSNK2B	0.32

CCNK	0.32
BCS1L	0.32
TMEM5	0.32
ANKRD46	0.32
ZBTB26	0.32
SRP9	0.32
GAR1	0.32
ZKSCAN5	0.32
C2CD3	0.32
COMMD5	0.32
PDAP1	0.32
TMEM189	0.32
ZSCAN12	0.32
GEMIN6	0.32
ZNF480	0.32
C6orf120	0.32
PSMA5	0.32
MBTD1	0.32
ZBTB10	0.32
FAM124A	0.32
GJC1	0.32
SMARCAD1	0.32
EIF3E	0.32
CDKN2AIPNL	0.32
PELP1	0.32
PSMA1	0.32
ENAH	0.32
CWF19L1	0.32
SAMD8	0.32
NUDT3	0.32
TUBE1	0.32
DNAJC14	0.32
MTA3	0.32
LRRC59	0.32
GOLT1B	0.32
C12orf4	0.32
ZNF7	0.32
ALG6	0.32
CNOT7	0.32
TSPYL5	0.32
C12orf45	0.32
PEX5	0.32
TXNDC16	0.32
C7orf13	0.32
GOLGA7	0.32
ZNF273	0.32
RAD23B	0.32
STRN3	0.32
PLEKHG5	0.32
NSMAF	0.32
NIPSNAP1	0.32
UQCRB	0.32
TMEM183A	0.32
PKN2	0.32
PEX3	0.32
MLLT10	0.32
KIAA0317	0.32
FAM189B	0.32

BOD1	0.32
ZMYND19	0.32
RRP1	0.32
ALG8	0.32
GAPDH	0.32
HEATR2	0.32
HNRNPA1L2	0.32
PRKD3	0.32
TTC35	0.32
SUB1	0.32
CWC27	0.32
PGM2	0.32
MARCKS	0.32
SRPRB	0.32
SNHG10	0.32
SPATA5	0.32
CHD8	0.31
CDC25B	0.31
CCDC52	0.31
SUZ12P	0.31
SNCAIP	0.31
SQLE	0.31
C11orf57	0.31
NDUFB3	0.31
CCDC75	0.31
MTO1	0.31
NAB1	0.31
CA8	0.31
FAM168A	0.31
ASF1A	0.31
SUMO1P3	0.31
HRSP12	0.31
RELL2	0.31
RCN2	0.31
DRG2	0.31
STAG2	0.31
C10orf84	0.31
IPO8	0.31
LASS6	0.31
PSMC6	0.31
SCAMP5	0.31
EIF3M	0.31
FUCA2	0.31
SAC3D1	0.31
FUBP1	0.31
PARS2	0.31
MAPK1	0.31
NUPL2	0.31
RBBP8	0.31
SBK1	0.31
CD3EAP	0.31
GNB1L	0.31
FANCF	0.31
IQCB1	0.31
DPH5	0.31
TBCA	0.31
AIMP1	0.31
MGAT4A	0.31

AKAP5	0.31
ALG10	0.31
RBM27	0.31
GSK3A	0.31
GPR89A	0.31
UBE2D2	0.31
C17orf76	0.31
PA2G4P4	0.31
ZNF384	0.31
PKP2	0.31
ZFAND1	0.31
RPRD1A	0.31
TMEM135	0.31
KCTD3	0.31
SLC25A5	0.31
TCF12	0.31
IREB2	0.31
PCLO	0.31
C1orf52	0.31
RRS1	0.31
USP45	0.31
SUV420H2	0.31
TMEM22	0.31
RRP12	0.31
ACVR2B	0.31
WDR18	0.31
CEP57	0.31
CRIPT	0.31
VTI1A	0.31
ICT1	0.31
NAA40	0.31
NARF	0.31
SLC25A17	0.31
SLC25A11	0.31
KIAA1598	0.31
ZNF749	0.31
MEA1	0.31
CPSF1	0.31
JMJD6	0.31
EIF2A	0.31
KLHDC5	0.31
RHOBTB3	0.31
ATE1	0.31
COX8A	0.31
RPRD1B	0.31
CAMSAP1	0.31
FAM122B	0.31
CBWD1	0.31
RFFL	0.31
RAP1GAP2	0.31
RTN3	0.31
FADS2	0.31
ZSCAN2	0.31
SHFM1	0.31
PABPC1	0.31
DAD1	0.31
ATAD1	0.31
PCK2	0.31

TMEM69	0.31
CHN2	0.31
VPS54	0.31
LOC728554	0.31
SF3B3	0.31
HMGB3	0.31
B4GALT6	0.31
C3orf1	0.31
LRRC16A	0.31
PGM3	0.31
AMD1	0.31
UBAC1	0.31
TOP1MT	0.31
MUTYH	0.31
FOXRED1	0.31
RMND5A	0.31
PSMD6	0.31
ID2	0.31
C20orf117	0.31
HNRNPU	0.31
UTP15	0.31
POLR1C	0.31
SDHB	0.31
ALKBH1	0.31
THAP10	0.31
FAM102B	0.31
USP10	0.31
ZMYM1	0.31
EIF5	0.31
PALB2	0.31
CTU1	0.31
CAMKK2	0.31
YKT6	0.31
COX4NB	0.31
PMS2L1	0.31
CEP250	0.31
B4GALNT1	0.31
C1orf174	0.31
PRPS2	0.31
MITD1	0.31
IGFBP5	0.31
ANAPC10	0.31
LEO1	0.31
MTMR4	0.31
RFC1	0.31
SNRNP25	0.31
ZKSCAN3	0.31
ZNF207	0.31
PGAM2	0.31
C7orf46	0.31
FLVCR1	0.31
PSMC2	0.31
VDAC1	0.31
CTCF	0.31
UBE2D1	0.31
ODF2	0.31
FAM83G	0.31
RCE1	0.31

CNP	0.31
TRAPPC9	0.3
CELSR3	0.3
TMEM132A	0.3
TXNRD1	0.3
LOC150786	0.3
SYT11	0.3
BRMS1L	0.3
PANK2	0.3
RPP25	0.3
VMA21	0.3
NUP93	0.3
ERMP1	0.3
HACE1	0.3
RAB23	0.3
7-Mar	0.3
B3GALNT1	0.3
HSPA9	0.3
ZNF71	0.3
LOC152217	0.3
VANGL1	0.3
RAB2A	0.3
TRMT11	0.3
FBXO22OS	0.3
XPNPEP3	0.3
VPS72	0.3
MMADHC	0.3
MATR3	0.3
C10orf46	0.3
TTC39C	0.3
DPP3	0.3
FOXA1	0.3
WTAP	0.3
LSM1	0.3
KHDC1	0.3
WAPAL	0.3
FOXN2	0.3
NCOA6	0.3
AVL9	0.3
C16orf63	0.3
PAPOLG	0.3
PUS3	0.3
NOVA1	0.3
PIGU	0.3
TMLHE	0.3
BRAF	0.3
RYK	0.3
SOX4	0.3
ARHGAP33	0.3
MYNN	0.3
RNF144A	0.3
RBM19	0.3
H19	0.3
MFSD2B	0.3
RABEP1	0.3
MLF2	0.3
RPS6KC1	0.3
COX6C	0.3

RAD1	0.3
NAP1L1	0.3
HMGCR	0.3
DGKE	0.3
KPTN	0.3
SNAPC3	0.3
ZNF410	0.3
NSL1	0.3
UTP14A	0.3
LSM7	0.3
NSUN2	0.3
PSMB1	0.3
BAZ1B	0.3
CCDC41	0.3
POLR2B	0.3
SNX6	0.3
SUV420H1	0.3
ARMC6	0.3
AHCTF1	0.3
TCHP	0.3
TUBB4	0.3
DDX20	0.3
ARL6	0.3
MYB	0.3
C14orf109	0.3
DDX27	0.3
C7orf70	0.3
CLCN2	0.3
ZNF518A	0.3
SUCLG1	0.3
HSPA8	0.3
C7orf28A	0.3
CNTLN	0.3
POLDIP2	0.3
ZDHHC13	0.3
SSR1	0.3
DPH3B	0.3
TBC1D7	0.3
FKBP9L	0.3
MRPS35	0.3
EXOSC5	0.3
CDK5RAP1	0.3
FBXO41	0.3
GTPBP10	0.3
CHCHD8	0.3
ZZZ3	0.3
KDM3A	0.3
TAOK1	0.3
FBXL14	0.3
COX10	0.3
GCFC1	0.3
SP4	0.3
PANK3	0.3
TRIM24	0.3
KLHL15	0.3
METTL6	0.3
TWISTNB	0.3
CBWD2	0.3

SAMD4B	0.3
NRD1	0.3
GMEB1	0.3
ZCCHC3	0.3
SMEK1	0.3
AATF	0.3
PKN1	0.3
UBA5	0.3
TMX2	0.3
ZBTB5	0.3
C12orf65	0.3
TIMM17A	0.3
C1orf131	0.3
SIKE1	0.3
EYA3	0.3
KCMF1	0.3
SMYD2	0.3
ZBTB9	0.3
ZNF569	0.3
PRPF38A	0.3
SGK196	0.3
