# Supplementary material for: DSCC1 interacts with HSP90AB1 and promotes the progression of lung adenocarcinoma via regulating ER stress
Source: Cancer Cell Int. 2023 Sep 23;23:208. doi: 10.1186/s12935-023-03047-w (PMC10518103; doi:10.1186/s12935-023-03047-w)
Supplement: Supplementary file 5 — Additinal File 5: Supplementary Table 3: Primers used in Quantitative PCR analysis [file 12935_2023_3047_MOESM5_ESM.pdf]

Primers used in Quantitative PCR analysis

Gene	Forward Primer	Reverse Primer
SOX2	5′- CATCACCCACAGCAAATGAC-3′	5′- CAAAGCTCCTACCGTACCACT-3′
NANOG	5′- GCAGGCAACTCACTTTATCC-3′	5′- CCCACAAATCACAGGCATAG-3′
4-Oct	5′ -GAGTGAGAGGCAACCTGGAG-3′	5′ -GCCGGTTACAGAACCACACT-3′
C-MYC	5′ - GGGCTTTATCTAACTCGCTGTA-3′	5′ -GCTATGGGCAAAGTTTCGTG-3′
CD133	5′- GGCCCAGTACAACACTACCAA-3′	5′- ATTCCGCCTCCTAGCACTGAA-3′
GAPDH	5′-GAGTCAACGGATTTGGTCGT-3′	5′-TTGATTTTGGAGGGATCTCG-3′
DSCC1	5′ -AAAGTTCCTTTGAACACATGCCT-3′	5′ -CGCATTCTGAAGTAGCATTCGT-3′
